# Hybrids Provide More Options for Fine-Tuning Flowering Time Responses of Winter Barley

**DOI:** 10.3389/fpls.2022.827701

**Published:** 2022-03-22

**Authors:** Miriam Fernández-Calleja, Francisco J. Ciudad, Ana M. Casas, Ernesto Igartua

**Affiliations:** ^1^Department of Genetics and Plant Production, Aula Dei Experimental Station - Spanish National Research Council (EEAD-CSIC), Zaragoza, Spain; ^2^Agricultural Technology Institute of Castilla and León (ITACYL), Valladolid, Spain

**Keywords:** vernalization sensitivity, adaptation, gene action, hybrid breeding, preanthesis phenological phases

## Abstract

Crop adaptation requires matching resource availability to plant development. Tight coordination of the plant cycle with prevailing environmental conditions is crucial to maximizing yield. It is expected that winters in temperate areas will become warmer, so the vernalization requirements of current cultivars can be desynchronized with the environment’s vernalizing potential. Therefore, current phenological ideotypes may not be optimum for future climatic conditions. Major genes conferring vernalization sensitivity and phenological responses in barley (*Hordeum vul*gare L.) are known, but some allelic combinations remain insufficiently evaluated. Furthermore, there is a lack of knowledge about flowering time in a hybrid context. To honor the promise of increased yield potentials, hybrid barley phenology must be studied, and the knowledge deployed in new cultivars. A set of three male and two female barley lines, as well as their six F_1_ hybrids, were studied in growth chambers, subjected to three vernalization treatments: complete (8 weeks), moderate (4 weeks), and low (2 weeks). Development was recorded up to flowering, and expression of major genes was assayed at key stages. We observed a gradation in responses to vernalization, mostly additive, concentrated in the phase until the initiation of stem elongation, and proportional to the allele constitution and dosage present in *VRN-H1*. These responses were further modulated by the presence of *PPD-H2*. The duration of the late reproductive phase presented more dominance toward earliness and was affected by the rich variety of alleles at *VRN-H3*. Our results provide further opportunities for fine-tuning total and phasal growth duration in hybrid barley, beyond what is currently feasible in inbred cultivars.

## Introduction

Higher and more stable crop yields are the main targets for cereal breeders. This goal is increasingly challenging in temperate regions, where major crops face growing threats from the impact of climate change, particularly from drought and heat events at critical developmental milestones during the crop cycle (Olesen et al., 2010; Porter et al., 2014; Trnka et al., 2014). Tight coordination of plant cycle to environmental conditions to match resource availability with the most sensitive growth stages is crucial for crop adaptation (Craufurd and Wheeler, 2009), and has a major effect on yield (Bolaños and Edmeades, 1993; Evans, 1996; González et al., 1999; Cockram et al., 2007; Tondelli et al., 2014; Flohr et al., 2018; Wiegmann et al., 2019). In this context, the current variety formats for cultivation should be re-assessed, as they may no longer be the highest-yielding ones. Further research on crop plasticity is necessary to adapt cereal crops to the range of future climatic conditions (Fatima et al., 2020). Winters in the temperate zone are projected to be warmer, so the vernalization requirement of current winter cultivars may be excessive, i.e., may not be met on time, due to a lower vernalizing potential of the environment (Saadi et al., 2015; Yang et al., 2019). Future ideotypes will have to combine specific vernalization and photoperiod responses fine-tuned to the projected climatic conditions prevalent for each region (Stratonovitch and Semenov, 2015; Gouache et al., 2017; Tao et al., 2017). Allelic variation at the *VRN-H1* gene already induces a gradation of vernalization needs to the barley plants, which have had a large impact on barley adaptation to regional climates (Casao et al., 2011b; Contreras-Moreira et al., 2019). Breeders must aim at deploying appropriate phenology gene combinations to optimize the crop foundation phase (vegetative and early reproductive), and construction phase (late reproductive) growth periods, as well as avoiding abiotic stresses at critical developmental stages, thus optimizing yield potential in target environments (Gouache et al., 2017).

Nowadays, there is growing interest in breeding hybrid cereal varieties, including barley. Hybrids have shown greater yield potential than inbred lines, due to exploitation of heterosis, greater yield stability under fluctuating environmental conditions, and the ease of pyramiding strategic combinations of dominant major genes (Longin et al., 2012; Mühleisen et al., 2013, 2014). Therefore, optimizing phenology in hybrid cultivars is a strategy to improve yields under current and future climate conditions. However, there is a lack of knowledge about flowering time gene action in a hybrid context.

Flowering time in barley is tightly regulated by genetic networks that respond predominately to day-length (photoperiod) and prolonged exposure to cold temperature (vernalization). Barley is a facultative long-day plant, flowering earlier under increasing day-lengths, and characterized by two major growth types, namely, winter and spring. Winter barleys need vernalization for timely flowering (Campoli and von Korff, 2014).

Vernalization genetic control is based on the epistatic system composed of flowering inducer *VRN-H1* (Trevaskis et al., 2003; Yan et al., 2003) and repressor *VRN-H2* (Yan et al., 2004). *VRN-H1* corresponds to gene *HvBM5A*, an orthologue of MADS-box *AP1* from *Arabidopsis* (Trevaskis et al., 2007), whereas *VRN-H2* has no clear correspondence in *Arabidopsis*. Winter barleys carry the functional dominant *VRN-H2* allele, accompanied by a cold-sensitive *VRN-H1* allele. Activation of *VRN-H1* is quantitative, with long cold treatments inducing higher levels of expression (von Zitzewitz et al., 2005; Sasani et al., 2009), which results in an earlier transition to the reproductive phase (Sasani et al., 2009). It presents a large number of alleles, which are defined by the length of the first intron (11 kb in the wild-type *vrn-H1*), and present a gradation of responses to vernalization (Takahashi and Yasuda, 1971; Szűcs et al., 2007), roughly proportional to the first intron length (Szűcs et al., 2007; Hemming et al., 2009; Casao et al., 2011a; Oliver et al., 2013; Guerra et al., 2021). Regarding the gene action of the *VRN-H1* allelic series, the accepted model states that the winter allele is recessive, while the rest are dominant (Takahashi and Yasuda, 1971; Haas et al., 2020). *VRN-H3* (*HvFT1*) is the key flowering inducer that integrates the photoperiod and vernalization pathways (Yan et al., 2006; Faure et al., 2007; Kikuchi et al., 2009), whose expression is induced under long-day conditions and promotes flowering (Turner et al., 2005; Hemming et al., 2008), and is an orthologue of *FT1* in *Arabidopsis* (Yan et al., 2006). Ample allelic variation at *VRN-H3* has been described, arising from sequence polymorphisms in the promoter and first intron (Yan et al., 2006; Hemming et al., 2008; Casas et al., 2011, 2021), and copy number variation (Nitcher et al., 2013; Loscos et al., 2014), as summarized in Fernández-Calleja et al. (2021), but there is no information on its gene action. According to the currently accepted model, during autumn, when temperate cereals germinate, *VRN-H2* represses *VRN-H3* expression. During winter, vernalization induces *VRN-H1* expression, resulting in *VRN-H2* repression in leaves and, consequently, activation of *VRN-H3* transcription in spring, which promotes the transition from the vegetative to the reproductive stage (Trevaskis et al., 2006; Distelfeld et al., 2009). At the whole plant level, this transition is visible as the appearance of the first node at the main stem, and the beginning of stem elongation (jointing stage). Besides the *VRN* genes, *HvODDSOC2* also plays a repressor role in the vernalization pathway (Greenup et al., 2010). This gene is the monocot orthologue of *Arabidopsis thaliana FLOWERING LOCUS C* (*FLC*, Ruelens et al., 2013). It is downregulated by prolonged cold exposure, was identified as a binding target of the VRN1 protein in barley, together with *VRN-H2* and *VRN-H3* genes (Deng et al., 2015), and plays a repressor role in absence of full vernalization (Monteagudo et al., 2019b). Genes *PPD-H1* and *PPD-H2* control photoperiod sensitivity. *PPD-H1* (*HvPRR37*, orthologue of *PRR7* in *Arabidopsis*) is the major determinant of long photoperiod response in barley (Turner et al., 2005). Its activity causes an increased expression of *VRN-H3* after vernalization fulfillment, promoting flowering under long-day conditions (Turner et al., 2005; Campoli et al., 2012; Mulki and von Korff, 2016). *PPD-H2* (*HvFT3*), which belongs to the FT gene family, induces early reproductive development in short-day conditions, or even long-day conditions when vernalization requirements have not been fully satisfied (Laurie et al., 1995; Faure et al., 2007; Casao et al., 2011a,b; Mulki et al., 2018). Phylogeographic and genetic studies suggest an adaptive role for this gene in winter barleys (Kikuchi et al., 2009; Casao et al., 2011b).

Barley breeding for the near future requires understanding the genetic mechanisms of adaptation, including vernalization responses, in a hybrid context. This work is intended to explore the inheritance and effect of several major flowering genes in heterozygosis, and their dynamics in relation to insufficient vernalization. For this purpose, an experiment with different vernalization treatments was designed aiming to evaluate the phenology and gene expression of key genes in the plant cycle duration, in a set of hybrid barleys and their parents. Here we show that hybrid combinations extend the available catalog of genetic responses to vernalization, opening new possibilities for optimizing phenology to specific areas using hybrids.

## Materials and Methods

### Plant Material

In this study, eleven barley (*Hordeum vulgare* L.) genotypes were used: two female parents (Female A and Female B), three pollinators (Male 1, Male 2, and Male 3), and six hybrids derived from their crosses (Hybrid A1, Hybrid A2, Hybrid A3, Hybrid B1, Hybrid B2, and Hybrid B3). The female parents are cytoplasmic male sterile (CMS) inbred lines used in the development of 6-row winter barley hybrids for Europe by Syngenta^®^ (Basel, Switzerland). The pollinators are advanced inbred lines developed in the framework of the Spanish Barley Breeding Program (Gracia et al., 2012), well adapted to the Mediterranean conditions, and without fertility restorer genes. The resultant offspring are male-sterile hybrids (female hybrids, F_1_F), an intermediate step in the production of a three-way hybrid, after further crossing with a fertility restorer genotype. The genotypes studied present different *VRN-H1* alleles, which are defined by the length of the first intron of the gene, and have different vernalization requirements, ranking from low to high cold needs. These alleles are *VRN-H1-4*, which presents a vernalization requirement of around 2 weeks, *VRN-H1-6* requires approximately 30 days, and *vrn-H1* requires not less than 7 weeks (Casao et al., 2011a). These alleles represent the main allelic diversity that is spread across Western European six-row winter barley. The strict winter allele (*vrn-H1*) prevails in North-western European barleys, whereas alleles *VRN-H1-4* and *VRN-H1-6* correspond to the two largest germplasm groups found in Spanish barley landraces (Casao et al., 2011a). The geographical distribution of these two alleles coincides with the harshness of winters in the Iberian region (Yahiaoui et al., 2008; Casao et al., 2011a; Contreras-Moreira et al., 2019). *VRN-H1-4* predominates in Southern and coastal Spanish landraces, while *VRN-H1-6* is frequently found in the continental inlands of Spain. Besides, the genotypes studied also present different alleles at other major genes involved in the control of vernalization responses and day-length sensitivity (Table 1).

**TABLE 1:** Genotypes for the genes associated with responses to vernalization and photoperiod in the parent lines under study.

	Vernalization, photoperiod, and				
	*earliness per se* genes				
Parent lines	*VRN-H1^a^*	*VRN-H2^b^*	*VRN-H3^c^*	*PPD-H1^d^*	*PPD-H2^e^*
Female A	*vrn-H1*	*VRN-H2*	*vrn-H3d(1)*	*PPD-H1*	*ppd-H2*
Female B	*VRN-H1-6*	*VRN-H2*	*vrn-H3a(1)*	*PPD-H1*	*ppd-H2*
Male 1	*VRN-H1-4*	*VRN-H2*	*vrn-H3d(1)*	*PPD-H1*	*ppd-H2*
Male 2	*VRN-H1-6*	*VRN-H2*	*vrn-H3c(1)*	*PPD-H1*	*ppd-H2*
Male 3	*vrn-H1*	*VRN-H2*	*vrn-H3a(1)*	*PPD-H1*	*PPD-H2*

*^a^Alleles based on the size of intron 1, following Hemming et al. (2009).*

*^b^Presence (dominant)/absence (recessive) of HvZCCT, following Karsai et al. (2005).*

*^c^Alleles based on two indels in the first 550 bp upstream of the start codon, two SNPs in intron 1, and CNV, coded as in Fernández-Calleja et al. (2021). vrn-H3a(1) = promoter deletion-insertion, intron 1 AG, CNV 1 copy, vrn-H3c(1) = deletion-insertion, TC, 1 copy, vrn-H3d(1) = insertion-deletion, TC, 1 copy.*

*^d^Alleles based on SNP22 of Turner et al. (2005), dominant = G, recessive = T.*

*^e^Presence (dominant)/absence (recessive), as in Faure et al. (2007).*

### Plant Growth Conditions, Phenotyping, and Sampling

A study under controlled conditions was designed to assess differences in development and gene expression in key genes controlling the duration of the plant cycle, after three vernalization treatments: complete (8 weeks of cold period, V8), moderate (4 weeks, V4), and low (2 weeks, V2).

Genotypes were exposed to treatments of 14 (low), 28 (intermediate), and 58 days (full vernalization) at 6 ± 2°C, under a short-day regime (8 h light/16 h dark). After vernalization, seedlings were transferred to a growth chamber with conditions set to long photoperiod (16 h light/8 h night), 220 μmol m^–2^s^–1^ light intensity, and 20°C day/16°C night temperatures. The duration of the vernalization treatments was set according to previous experiments. Fourteen days are enough for genotypes carrying the *VRN-H1-4* allele, 58 days are sufficient for cultivars with strict winter growth habits, and 28 days is an intermediate condition.

The study was carried out in two stages, growing plants independently for gene expression and phenotyping, and using the same growth chamber. For phenotyping, plants were grown in trays of 12 cells (650 cc per cell) from sowing until flowering. The number of days to the appearance of the first node at the base of the main stem, or stage 31 on the Zadoks scale (Z31), and the number of days to awn tipping or Z49 (Zadoks et al., 1974) were recorded. Four plants were assessed for each genotype and treatment. After discarding dead plants and outliers, data from the best three plants per treatment were kept. The phenotyping experiment lasted 130 days.

The gene expression experiment was carried out in two batches due to growth chamber capacity, split according to the females, due to space limitations. One subset comprised the hybrids derived from Female A and respective parents (Batch A), and the other subset included the hybrids coming from Female B and respective parents (Batch B). Therefore, the three male parents were present in the two batches. Plants were grown in trays of 35 cells (200 cc per cell), from sowing until sampling. In the second stage (phenotyping), undisturbed plants from all eleven genotypes were simultaneously assessed for developmental traits.

The last expanded leaf of four independent plants was sampled 17 and 35 days after the entry in the growth chamber (i.e., after the end of the respective vernalization treatment), 14 h into the light period (2 h before the end of the day). Samples were frozen in liquid nitrogen, homogenized (Mixer Mill model MM400, Retsch, Haan, Germany) and conserved at −80°C until RNA isolation. In principle, three plants were analyzed. If they were clearly dissimilar, the fourth plant was also analyzed, and the best three were kept for further analysis.

### Gene Expression Analysis

Using the Total RNA Mini Kit for Plants (IBI Scientific, Dubuque, IA, United States) RNA extraction was carried out following manufacturer instructions. Total RNA (1 μg) was employed for cDNA synthesis using SuperScript III Reverse Transcriptase (Invitrogen, Carlsbad, CA, United States) and oligo (dT) 20 primer (Invitrogen, Carlsbad, CA, United States). RT PCR quantification (ABI 7500, Applied Biosystems, Waltham, MA, United States) was performed for samples from each time point and vernalization treatment. Four plants (biological replicates) per sampling time, treatment, and genotype were sampled. Three biological replicates and two technical replicates were tested per sample and pair of primers (*VRN-H1*, *VRN-H2*, *VRN-H3*, *PPD-H1*, *HvODDSOC2*, and *PPD-H2*). When outliers were detected, the fourth plant was also analyzed, and the three replicates which showed the best agreement were kept. Primer sequences and conditions are specified in Supplementary Table 1. Gene expression levels were normalized to *Actin* expression, considering primer efficiencies. The average of the two technical replications of ΔCt (Ct *Actin*–Ct target gene) was used as the experimental unit for statistical analyses, to protect against small pipetting errors.

### Statistical Analysis

Statistical analyses were carried out using R software (R Core Team, 2013). Differences in Z31, Z49, and the lag between the latter stages (Lag Z31–Z49) between genotypes and treatments were evaluated using the ANOVA procedure in R. The ANOVA model included genotype, vernalization treatment, and genotype by treatment interaction, all taken as fixed factors. The three plants sampled per genotype were considered biological replicates. The genotype factor was broken down into the most informative contrasts: hybrids *vs.* parents, females *vs.* males, and finally, hybrids from Female A *vs.* hybrids from Female B. Multiple comparisons were obtained by Fisher’s protected least significant differences (LSD) with the R package “emmeans” (Lenth et al., 2018).

Using the same statistical procedure, differences in vernalization sensitivity for Z31, Z49 and lag Z31–Z49 between genotypes and treatments (V8–V4, V4–V2, and V8–V2) were tested. Vernalization sensitivity comparing V8 and V4 treatments was calculated by subtracting the value for an individual observation in the 8-week vernalization treatment from the mean value of that genotype in the 4-week vernalization treatment, for each suitable variable. The same procedure was followed for each variable considering the V4–V2 or V8–V2 treatments.

For gene expression results, Batch A and Batch B were analyzed separately. The ANOVA model included genotype, treatment, sampling time, and factorial interactions. The analyses of variance were performed considering all factors (genotype, sampling time, and treatment) as fixed. The three biological replications were considered replicates. The contrasts defined were hybrids *vs.* parents, hybrids *vs.* females, hybrids *vs.* males, and females *vs.* males. Means were compared using the LSD test (*P* < 0.05).

A correlation network analysis was carried out with the R package “qgraph” (Epskamp et al., 2012). We performed a multiple factorial analysis (Pagès, 2002) using R packages “FactoMineR” (Lê et al., 2008) and “factoextra” (Kassambara and Mundt, 2017). This method summarizes and displays a complex data table in which individuals are described by several sets of variables (quantitative and/or qualitative) structured into groups. It requires balancing the influences of each set of variables. Therefore, the variables are weighted during the analysis. Variables in the same group are normalized using the same weighting value, which can vary from one group to another. In our case, we summarized the observations described by a set of variables structured into four groups (*Treatment*, *Genotype*, *Development*, and *Gene expression*). *Genotype* and *treatment* are groups based on categorical variables specifying the genotype identity of each individual and the vernalization treatment to which they were subjected. *Development* and *gene expression* quantitative variables were considered as active groups and their contribution was considered to define the distance between individuals. Each variable within a group was equally weighted, so the influence of each set of variables in the analysis was balanced.

## Results

**FIGURE 1:**
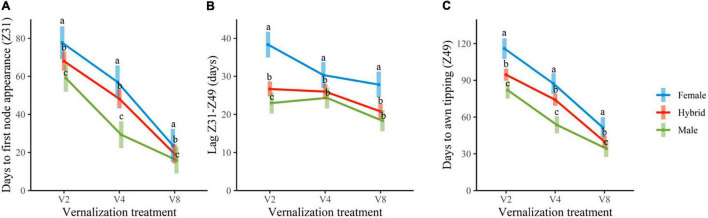
From left to right, the average duration of time until Z31, **(A)**, lag Z31–Z49 **(B)**, and total time until Z49 **(C)**, for the three groups of genotypes, male parents, female parents, and hybrids. Error bars are 95% CIs. For each developmental variable and treatment, group means with a different letter are significantly different at *P* < 0.05 according to the contrasts performed for the overall ANOVA.

**FIGURE 2:**
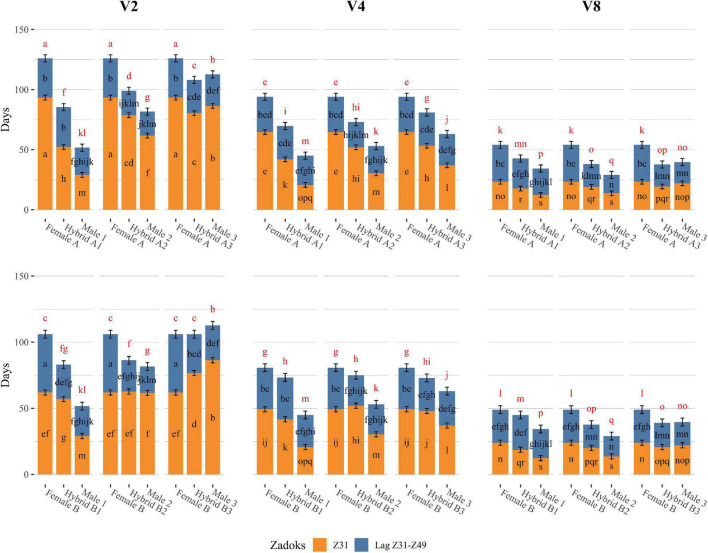
Developmental differences of triads of genotypes (Female, Hybrid, and Male) grown under 16 h light, in response to different vernalization treatments (V2: 2 weeks of vernalization, V4: 4 weeks of vernalization, V8: 8 weeks of vernalization; 4–8°C, 8 h light). Days to first node appearance (Z31) are represented as the height of the orange bars, days to awn tipping (Z49) are represented as the total height of the bars, and the lag period between Z31 and Z49 (LagZ31–Z49) is represented as the segment in blue. Genotypes from Batch A are shown in the upper part of the graph, whereas genotypes from Batch B are shown in the bottom part of the graph. Each of the three columns of facets represents a vernalization treatment, low on the left, moderate in the middle, and complete on the right. Each subplot within each facet contains one triad of genotypes composed of one female parent on the left, the male parent on the right, and the hybrid in the middle. Error bars are 95% CIs for Z31 and LagZ31–Z49. For each developmental variable, bars with a different letter are significantly different at *P* < 0.05. Letters in red represent the comparison of the means for Z49.

Insufficient vernalization markedly extended the growth cycle of plants. At the complete vernalization treatment (V8), the duration of the two phases considered (time until Z31 and lag Z31–Z49) was rather similar. With increasingly insufficient vernalization, time to awn tipping (Z49) raised progressively. Most of this lengthening occurred in the period until the first node appearance (Z31), although additional delays were observed in the late reproductive phase (lag Z31–Z49), particularly for the female genotypes (Figures 1, 2).

### Vernalization Response of Parent and Hybrid Genotypes

Differences in the length of developmental phases between genotypes were also detected (Supplementary Tables 2, 6). Male parents (the Mediterranean adapted) were earlier than female parents. In general, hybrids showed an intermediate phenotype between both parents for days to reach the jointing stage (Z31), and days to awn tipping in all vernalization treatments. However, the length of the late reproductive phase of the hybrids was closer to that of the male parents (Figures 1, 2 and Supplementary Table 3).

Parents and hybrids presented different vernalization responses (Figure 2). This differential behavior was mostly explained by changes in the duration of the phase until jointing, which was associated with the *VRN-H1* allele present (negative correlation indicated in Supplementary Figure 1), and their dosage in the case of the hybrids. Increasing vernalization treatments minimized these differences between genotypes, especially in those triads (female, hybrid, male) where different vernalization alleles were crossed (Figure 2).

With complete vernalization (V8), all genotypes reached the Z31 stage in a similar range, although some differences were still evident. Male 1 and Male 2, carrying low (*VRN-H1-4*) and medium (*VRN-H1-6*) vernalization requirement alleles, were the earliest until Z31. Winter parents (*vrn-H1*), Male 3 and Female A, and Female B (*VRN-H1-6*), showed intrinsic lateness, reaching the Z31 stage 10 days later than the other male parents did. All six hybrids reached first node appearance at approximately the same time, independently of their *VRN-H1* allele, and significantly earlier than their female parents (Figure 2).

When vernalization was moderate (V4), the differences in days to first node appearance between genotypes were accentuated (Figure 2). Male 1 (*VRN-H1-4*) maintained a short Z31 phase, only 8 days longer compared to V8. Male 2 (*VRN-H1-6*) and Male 3 (*vrn-H1*) were more affected by the reduction of the cold treatment, although they only delayed their Z31 date around 15 days compared to the V8 treatment. The female parents, in contrast, experienced a remarkable delay in days to first node appearance, particularly large for the winter Female A (40 days, Supplementary Figure 2). Interestingly, both Male 3 and Female A carry the strict winter *vrn-H1* allele, but they differed almost 30 days at Z31 for the V4 treatment (Figure 2). This finding indicates that early alleles of Male 3 at genes other than *VRN-H1* are having a shortening effect on the Z31 phase. This gene could be *PPD-H2*, as will be discussed later. For hybrids, in general, we observed intermediate phenotypes between the behaviors of their parents. Hybrids A1 and B1, carrying one copy of the *VRN-H1-4* allele, were the least affected by the reduction in the cold treatment, reaching Z31 earlier than any other hybrid. Hybrids from Male 2 and Male 3 showed a longer delay in Z31 with reduced vernalization (Figure 2).

When vernalization was low (V2), the Z31 phase was prolonged by 15 days or more for most genotypes (compared to V4). The exception was the genotypes carrying the *VRN-H1-4* allele, which reached Z31 considerably earlier than the rest of the genotypes (Figure 2) and had a low vernalization sensitivity (Supplementary Figure 2). Winter genotypes suffered the largest changes in time until Z31 when comparing V4 and V2, particularly Male 3 and its crosses. The differences between the females were even more pronounced in this treatment (V2), Female A reached Z31 30 days later than Female B (Figure 2). This agrees with the alleles that these genotypes carry at *VRN-H1*. Female A carries the winter allele (*vrn-H1*), characterized by a higher sensitivity to vernalization than the *VRN-H1-6* allele of female B. This dissimilarity in Z31 dates between females translated into differences in Z31 duration also between A and B hybrids (Figure 2). For instance, Hybrid A2 (*vrn-H1*/*VRN-H1-6*) delayed the jointing stage 16 days more than Hybrid B2 (*VRN-H1-6*/*VRN-H1-6*) when reducing the vernalization treatment from 4 to 2 weeks (Supplementary Figure 2), indicating a dosage effect of winter *VRN-H1* alleles. Particularly interesting in this treatment is the change in ranking observed in the slower developing parents. Male 3, which bears the active allele at *PPD-H2*, reached Z31 earlier than both female parents under moderate (V4) to complete (V8) vernalization. By contrast, at V2, Male 3 (*vrn-H1*) delayed significantly its early development, reaching the Z31 stage later than Female B (*VRN-H1-6*) and almost at the same time as Female A (*vrn-H1*) (Figure 2). The striking reduction in time to jointing of Male 3 and Hybrid B3 with moderate vernalization, but not in the low vernalization treatment, agrees well with the hypothesis that *PPD-H2* needs some cold to come into play (Monteagudo et al., 2019b).

**FIGURE 3:**
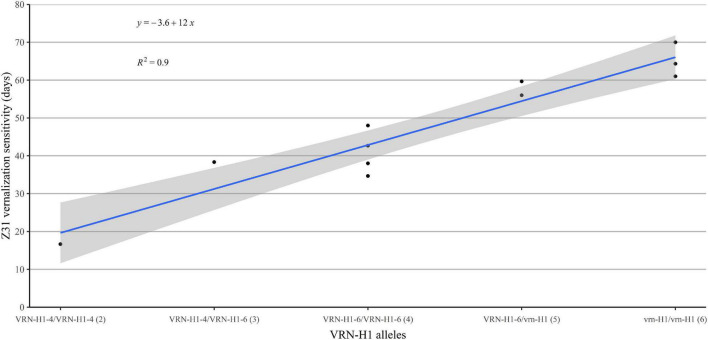
Regression of vernalization sensitivity (duration until Z31 at V2 minus duration until Z31 in V8) on the allelic constitution at *VRN-H1*, coded as 1 (*VRN-H1-4*), 2 (*VRN-H1-6*), or 3 (*vrn-H1*), proportional to the vernalization requirement induced by each allele. The number within brackets indicates the vernalization requirement score of the genotype, according to their *VRN-H1* alleles.

In summary, the Z31 phase was clearly the most sensitive period to the cold treatment. Vernalization sensitivity differed between genotypes (Supplementary Tables 2, 7), and seemed related to the *VRN-H1* allele present and proportional to the *VRN-H1* allele dosage. Genotypes carrying the low vernalization requirement allele *VRN-H1-4* reached the jointing stage earlier than the rest of the genotypes regardless of the vernalization treatment. In contrast, genotypes carrying winter *vrn-H1* alleles increased steeply the time to reach Z31 stage in the low vernalization treatment. Genotypes with a *VRN-H1-6* allele delayed jointing stage under insufficient vernalization, but not as much as strict winter types. We recoded *VRN-H1* alleles as a categorical variable with integer numbers roughly proportional to their associated vernalization response (1-2-3, for *VRN-H1-4*, *VRNH1-6*, and *vrn-H1*, respectively). We calculated a synthetic vernalization score by adding the values for the two alleles carried by each genotype. A regression of vernalization sensitivity (in this case, the difference V8–V2 for Z31) on the genotypic score produced a very good fit (Figure 3), supporting the dosage effect of *VRN-H1*.

The differences observed for the jointing phase were maintained until awn tipping but modulated by the effect of other genes on the late reproductive phase, likely *VRN-H3* and *PPD-H2* (Supplementary Figure 1). Male 1 and its crosses showed a constant and rather long lag Z31–Z49 phase across cold treatments. Conversely, Male 2 and its hybrids stood out for a consistent short late reproductive phase across treatments, probably because they carry the fast *vrn-H3c(1)* allele at *VRN-H3*. This additional precocity allowed them to be the fastest genotypes in reaching Z49 in V8, among parents and hybrids, respectively, even earlier than *VRN-H1-4* carriers. Male 3 and its crosses also showed a particularly short lag Z31–Z49 phase, but only at V8. Interestingly, female parents differed in their late reproductive phase duration patterns. Female A showed a constant duration of the late reproductive phase across treatments, whereas Female B showed a lag Z31–Z49 phase increasingly long with decreasing vernalization treatments (Figure 2). This observation was not translated into the hybrids, as they more closely resembled their male parents in the duration of the late reproductive phase.

Focusing on inheritance, hybrids derived from Male 1 and Male 2 showed an intermediate phenotype for Z31 and Z49, between the early male parents and the late female parents, in all vernalization treatments. Nevertheless, the lag Z31–Z49 phase of Male 2 hybrids was as short as that of their male parent (Figure 2), indicating the dominance of the Male 2 early allele controlling this phase. Hybrids A3 and B3, in contrast to the other hybrids, did not show a consistent intermediate phenotype between their parents. In the low and complete vernalization treatments, hybrids A3 and B3 headed Z49 as early as the early parent, due to a dominant short late reproductive phase similar to that of Male 3 (Figure 2).

### Gene Expression

Differences among genotypes were detected for the expression of all genes tested (Supplementary Tables 4, 5, 8, 9).

**FIGURE 4:**
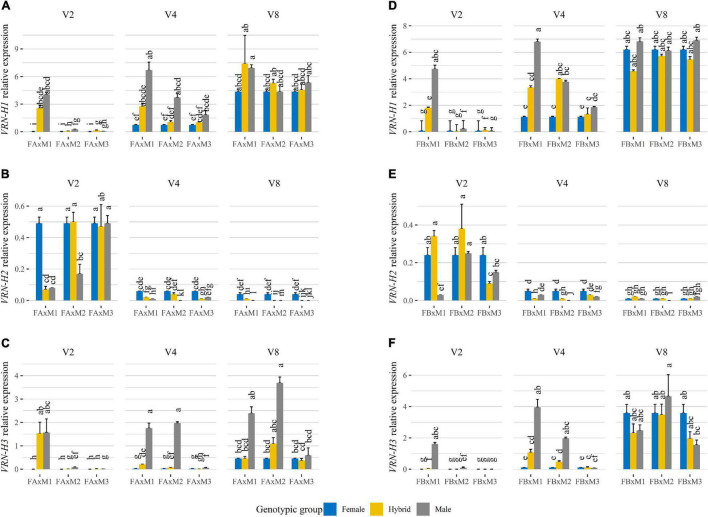
Relative expression levels, at 35 days of growth in each treatment, of *VRN-H1* **(A,D)**, *VRN-H2* **(B,E)**, and *VRN-H3* **(C,F)** assayed by qRT-PCR in triads of barley genotypes (Female, Hybrid, Male) grown under 16 h light, in response to different vernalization treatments (V2: 2 weeks of vernalization, V4: 4 weeks of vernalization, V8: 8 weeks of vernalization; 4–8°C, 8 h light). Plots **(A–C)** correspond to Female A crosses (Batch A). Plots **(D–F)** correspond to Female B crosses (Batch B). Each plot is divided into three facets, each of them containing gene expression assayed for one vernalization treatment, and the three triads of genotypes composed of one female parent in blue, the male parent in gray, and the hybrid in yellow. The triads are represented as abbreviations of the crosses between the parents, e.g., FA × M1: Female A × Male 1. The results shown are normalized to the level of the housekeeping gene *Actin* for each genotype and treatment. Mean of 3 biological replicates. Error bars represent the SEM. For each gene and batch, bars with a different letter are significantly different at *P* < 0.05, according to ANOVA that included genotypes and all treatments.

In all genotypes, *VRN-H1* expression increased gradually with increasing duration of vernalization (Figure 4 and Supplementary Figure 3), although differences between *VRN-H1* alleles were evident. Male 1 and its hybrids, carrying *VRN-H1-4*, were the only genotypes showing upregulated *VRN-H1* expression after just 2 weeks of vernalization. After vernalization for 4 weeks, *VRN-H1* expression also reached high levels in Male 2 and Hybrid B2, both carrying the medium vernalization requirement allele *VRN-H1-6*. The rest of the parents and hybrids required 8 weeks of cold to show high *VRN-H1* transcript levels (Figures 4A,D). However, not all differences in *VRN-H1* expression levels were due to allelic differences. For instance, both Female B and Male 2 carry the *VRN-H1-6* allele but present different *VRN-H1* expressions at V4 (Figures 4A,D). Hybrid B2 had the same (higher) *VRN-H1* expression as Male 2, indicating higher repression of *VRN-H1* in Female B, which is lost in the hybrid. In general, *VRN-H1* expression levels paralleled plant development patterns across genotypes and treatments.

When vernalization was complete, all parents and hybrids showed a high *VRN-H1* expression, probably caused by saturating vernalization requirements. With incomplete vernalization, however, *VRN-H1* expression in hybrids was, in general, intermediate between their parents (Figures 4A,D). Additive gene action caused by a dosage effect of *VRN-H1* was visible at the gene expression level, supporting the hypothesis that the duration of the phase until jointing is related to the effect of this gene. This was apparent when comparing Hybrid A2 (*vrn-H1*/*VRN-H1-6*), which did not peak until V8 (Figure 4A), with Hybrid B2 (*VRN-H1-6*/*VRN-H1-6*), in which *VRN-H1* expression peaked already with 4 weeks of vernalization (Figure 4D).

All lines carried the active *VRN-H2* allele, but differences in its expression were observed (Figures 4B,E). As expected, *VRN-H2* expression decreased with increasing duration of the vernalization treatment, and inversely correlated to the expression of *VRN-H1*. A similar trend was observed for *HvODDSOC2* (Supplementary Figures 4B,E). Nevertheless, there were some differences in *VRN-H2* expression among genotypes carrying the same winter *VRN-H1* allele. Indeed, at V4 and V8 treatments, the repression of *VRN-H2* in Male 3 and Hybrid A3 was higher than in the Female A (Figure 4B), despite all being winter types. This result agrees with an antagonistic relationship between *VRN-H2* and *PPD-H2* (present only in Male 3 and its hybrids).

With increasing duration of vernalization (Figures 4C,F), *VRN-H3* expression increased, and followed closely that of *VRN-H1*. There were no apparent differences in expression between *VRN-H3* alleles. Again, we could observe differences in expression among the two winter parents (Female A and Male 3), with Male 3 showing the highest *VRN-H3* expression in all vernalization treatments.

Across vernalization treatments and genotypes, *PPD-H1* expression was consistently high. All genotypes assessed in the experiment carry the photoperiod-sensitive *PPD-H1* allele (Supplementary Figure 4D).

Expression of *PPD-H2* was detected in all genotypes that carried the gene, i.e., Male 3, Hybrid A3, and Hybrid B3 (Supplementary Figures 4, 5). *PPD-H2* expression was detected after 4 and 8 weeks of cold, but not in the 2-week vernalization treatment, confirming that a cold period is needed to induce its expression in winter genotypes. Expression in Male 3 and Hybrid B3 in the V4 and V8 treatments was similar, indicating the dominance of the active *PPD-H2* allele (Supplementary Figure 5F).

### Associations Between Developmental Phases and Flowering Time Genes Expression

**FIGURE 5:**
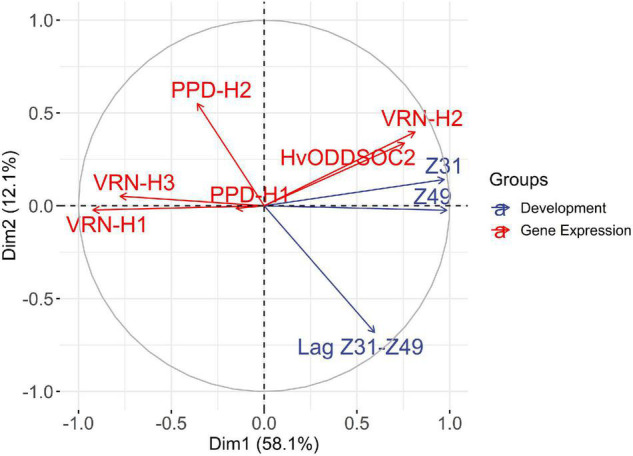
Multiple factorial analysis (MFA), variable correlation circle. The plot shows the correlation of the quantitative variables with the MFA axes. Variables related to developmental phases are depicted in blue and variables related to gene expression are depicted in red. The expression of flowering promoters *VRN-H1*, *PPD-H1* (ns), *VRN-H3*, and *PPD-H2* shows a negative correlation with the length of developmental phases. On the contrary, the expression of flowering repressors *VRN-H2* and *HvODDSOC2* shows a positive correlation with the length of developmental phases and negative correlations with the promoters, positioned in the opposite semicircle. The first dimension represents mainly the time to reach the jointing stage and the genes that influence it, whereas the second dimension is more related to the late reproductive phase.

We performed a multiple factorial analysis (MFA) to examine patterns of relationships between developmental phases and gene expression averaged over the two sampling dates (Supplementary Figure 6). The expression of flowering inducers *VRN-H1*, *PPD-H1* (ns), *VRN-H3*, and *PPD-H2* showed a negative correlation with the length of developmental phases (Figure 5 and Supplementary Figure 1), i.e., higher expression of gene inducers was related to earliness. On the contrary, the expression of flowering repressors *VRN-H2* and *HvODDSOC2* showed a positive correlation with the length of developmental phases and negative correlations with the flowering inducers, positioned in the opposite semicircle (Figure 5). The first dimension, accounting for almost 60% of the variance, summarized the time to reach the jointing stage and awn tipping, and the expression of the regulators involved in the control of the length of these periods, i.e., vernalization genes *VRN-H1* and *VRN-H2*, and *HvODDSOC2* and *VRN-H3*. The closeness of Z49 and Z31 vectors is explained by a correlation coefficient of 0.98 between the two variables. The duration of the lag Z31–Z49 had large loadings on the two dimensions and was relatively independent of the duration until Z31. The second dimension (12% of the variance) was related to the duration of the lag Z31-Z49, the expression of *PPD-H2*, and, to a lesser extent, *VRN-H2* and *HvODDSOC2*, reflecting the negative correlation between *PPD-H2* expression and the duration of the lag Z31–Z49. The variable *PPD-H1* was located close to the origin, indicating a poor representation on the factor map.

When plotting the individuals in the MFA, we could observe that the first axis mainly opposed the genotypes in the V8 and V2 treatments (Supplementary Figure 7). The genotypes Female A and Hybrid A3, carrying winter alleles at *VRN-H1*, showed the highest positive coordinates in the x-axis, which were positively correlated with a longer Z31 phase and a lower expression of *VRN-H1*. In contrast, genotypes characterized by a lower vernalization sensitivity, Male 1 and Male 2, showed the lowest negative coordinates, indicating shorter periods until the first node appearance and higher expression of *VRN-H1*. The second axis was essentially associated with genotypes Hybrid A2, Hybrid B3, Male 2, and Male 3, characterized by a short lag Z31–Z49 phase and low values of *VRN-H2* expression. On the opposite side of this axis, genotypes Female B and Hybrid A1 showed a consistently long late reproductive phase.

We detected variation in the length of the late reproductive phase, and its response to vernalization, which seemed related to the *VRN-H3* allele. We observed that those genotypes carrying the *vrn-H3c(1)* allele presented a short late reproductive phase independently of vernalization. Male 2 and its hybrids carry this allele and are represented by horizontal ellipses located on the positive side of the y-axis of the individuals’ MFA for genotypes (Supplementary Figure 8). In contrast, those genotypes carrying one or two copies of the *vrn-H3d(1)* allele (Female A, Male 1, and derived hybrids) showed a constant and long duration of the late reproductive phase across treatments, and their ellipses are located in the negative coordinates of the y-axis (Supplementary Figure 8). Besides, Male 1 and Hybrid A1 were represented by small ellipses, pointing out a reduced variance in their responses across vernalization treatments. In contrast, Hybrid B3 showed a more vertical distribution, indicating higher variance for the second axis, related to lag Z31–Z49 phase, *PPD-H2*, and *VRN-H2* expression (Supplementary Figure 8).

## Discussion

Climate change is challenging current agricultural practices, posing questions about the best combinations of genotype × environment × management options for the near future (Cooper et al., 2021). Sheehan and Bentley (2021) recently pointed out the need for greater flexibility in varietal flowering time to sustain United Kingdom wheat productivity, a view that can be easily extended to barley, and to other geographical areas. Other adaptation strategies that extend the catalog of possibilities include shifts in the sowing date. Recent studies suggest shifting toward earlier sowings to offset climate change impacts and increase cereal yields in the future scenario (Zheng et al., 2012; Hunt et al., 2019). Phenological adjustment of barley hybrids requires acquiring detailed knowledge of the functioning of major flowering time genes in heterozygosis. Our experiment supposes the first step in this direction. We exposed a set of hybrids and their parents to a range of vernalization conditions, which has provided some new insights for phenology management in hybrid barley breeding.

### Flowering Time in Hybrids Is Intermediate Between Parents

The Mediterranean-adapted lines used as pollinators were earlier than the central European elite lines used as female parents, whereas the hybrids were intermediate. This was true for both Z31 (jointing) and Z49 (awn tipping) stages, across all vernalization treatments, indicating the presence of additive inheritance. Additivity was not complete, however, as hybrids reached the Z31 stage significantly later than the average of parental lines (1.33 days, *p*-value < 0.001). In contrast, hybrids headed significantly earlier than the parental average (1.21 days, *p*-value < 0.01), due to a shorter late reproductive phase of hybrids, compared to the parental average (2.55 days, *p*-value < 0.001). The addition of the two phases resulted in slight heterosis toward earliness for Z49, which is a common finding in cereals, as reported for wheat (Borghi et al., 1988; Barbosa-Neto et al., 1996; Ahmed et al., 2000; Corbellini et al., 2002; Dreisigacker et al., 2005; Longin et al., 2013; Zhao et al., 2014; Al-Ashkar et al., 2020), triticale (Oettler et al., 2001), and barley (Zali and Allard, 1976; Oury et al., 2000; Bernhard et al., 2017). However, we revealed a distinct gene action at each developmental stage, with the prevalence of additivity in the foundation phase (up to jointing), and a trend toward dominance for earliness in the construction phase. This was not unexpected, as the different genetic control of the length of the preanthesis phenological phases in winter cereals is well supported by strong experimental evidence (Slafer and Rawson, 1994; Miralles and Richards, 2000; González et al., 2002; Gol et al., 2017; Ochagavía et al., 2018).

### Vernalization Mostly Affects the Foundation Growth Period, Which Is Controlled by Allelic Variation at *VRN-H1/VRN-H2* Genes

The vernalization treatments reduced the duration of the time until Z31 (72% on average, comparing V8 with V2), and lag Z31–Z49 (23% on average). Therefore, the sensitivity to vernalization mostly affected the vegetative and early reproductive phases, largely in agreement with the literature (Flood and Halloran, 1984; Griffiths et al., 1985; Roberts et al., 1988; Slafer and Rawson, 1994; Whitechurch et al., 2007), although strong effects of vernalization on the duration of the construction phase have also been reported (González et al., 2002). We observed this last effect only for the females.

Despite testing very different genotypes, the agreement between phenological development and gene expression supported our assumption that the range of responses to vernalization can largely be traced to the effect of the alleles present in *VRN-H1*. The multifactorial analysis indicated that the length until the reproductive transition (Z31) was associated with the pattern of expression of *VRN-H1* and *VRN-H2* genes, whose epistatic interaction controls the response to vernalization (von Zitzewitz et al., 2005). A novel finding of this study is that the length of the cold treatment needed to induce the expression of *VRN-H1*, as well as the degree of promotion toward flowering, depended on the allele constitution and dosage at *VRN-H1*. The dynamics of expression of *VRN-H1* alleles in the parents responded to the expectations of the gradual vernalization requirements induced by the three alleles. Thus, parents carrying the *VRN-H1-4* allele showed higher *VRN-H1* expression and accelerated development after just 2 weeks of cold; *VRN-H1-6* parents needed at least 4 weeks to reach the same point, whereas those carrying the *vrn-H1* allele required 8 weeks. The comparisons between homozygotes (parents and hybrids) indicated gradually decreasing vernalization requirements induced by alleles *vrn-H1*, *VRN-H1-6*, and *VRN-H1-4*, which confirms the gradation in the strength of flowering promotion displayed by the allelic series at *VRN-H1* (Takahashi and Yasuda, 1971; Szűcs et al., 2007; Casao et al., 2011a).

### Additive Inheritance of *VRN-H1* Winter Alleles

The use of parental lines with different *VRN-H1* alleles provided the opportunity to assess the gene action at this locus. When *VRN-H1* alleles were confronted in the crosses, we observed intermediate Z31 and Z49 phenotypes (and *VRN-H1* expression) between the early and late alleles indicating additivity of the effect of winter *VRN-H1* alleles. In triads where there was no variation for *VRN-H1*, the phenotypic differences between genotypes were small, regardless of the vernalization treatment, and most likely due to other genes.

The prevalent view among geneticists indicates the dominance of the spring growth habit over the winter type (Takahashi and Yasuda, 1971; Dubcovsky et al., 2005; Fu et al., 2005). This view is supported by a dominant inheritance of the gene *VRN-H1* at the expression level in spring × winter crosses (Haas et al., 2020). Our results challenge this view. In fact, a review of the literature finds other results in agreement with ours. Some studies found hybrids with intermediate flowering dates between parents carrying spring *VRN-H1* and winter *vrn-H1* alleles. While complete dominance may occur in particular environmental conditions, experiments covering a wider and more realistic range of conditions revealed that additivity is the rule more than the exception in the vernalization process (Kóti et al., 2006; Szűcs et al., 2007). The additivity in the inheritance of winter *VRN-H1* alleles has agronomic implications. It expands the range of barley flowering time and vernalization responses available using hybrid combinations and can be used by breeders to fine-tune varietal vernalization needs to the target environments.

A reduced vernalization requirement, matching winter harshness level, may cause timely flowering and enhance yield. In fact, earliness conferred by *VRN-H1-4* was associated with increased grain yield in warm sites prone to the occurrence of terminal water stress (Mansour et al., 2014). Therefore, it seems a good choice to deploy in barley breeding for future scenarios in which current vernalization potential will be reduced, either in homozygosis or in hybrid combinations with other alleles.

### The Construction Growth Period Shows a Dominant Inheritance Controlled by *FT*-Family Genes

Effects of *VRN-H1*, *VRN-H2*, and *PPD-H2* have been associated mainly with the length of the vegetative and early reproductive phases (Gol et al., 2017; Mulki et al., 2018). *VRN-H3* and *PPD-H1*, however, seem to affect the length of the late reproductive phase (Alqudah et al., 2014). In this experiment, *PPD-H2* expression apparently affected the duration of the jointing phase, but mostly the late reproductive phase, as also noticed by Casas et al. (2011).

We observed that the presence of *PPD-H2* modulated the responses of the *VRN-H1* alleles. In the two genotype triads involving a functional *PPD-H2* allele (including Male 3), we detected differences in the duration of development until the initiation of the jointing phase, which did not match the expectations based solely on their *VRN-H1* alleles. Male 3 and its hybrids showed a steeper reduction in development time, in response to moderate and complete vernalization than the female parents (both carrying a non-functional *ppd-H2* allele). However, this effect was absent in the low vernalization treatment: Female B (*VRN-H1-6*) was earlier at Z49 than Male 3 (*vrn-H1*) at V2, but this order was reversed at V4. Concurrent with this crossover of cycle duration, we detected *PPD-H2* expression, in the male or the hybrid, only after 4 and 8 weeks of cold, but not in the 2-week vernalization treatment. This suggests that *PPD-H2* responds not only to photoperiod, but also to vernalization, and helps to accelerate development only after some vernalization has occurred (between 2 and 4 weeks in this case). This result agrees with Monteagudo et al. (2019b) who found that *PPD-H2* expression required some developmental trigger (either a cold period or advanced plant age) in winter barleys. Moreover, the earliness effect of *PPD-H2* was highly conspicuous in the shortening of the late reproductive phase of the hybrid and male when fully vernalized. As a result, hybrids carrying *PPD-H2* flowered at least as early as the earlier parent when vernalization was fully satisfied, indicating the dominance of the *PPD-H2* functional allele.

Boosting the development by ensuring timely completion of the cycle, *PPD-H2* is predominant in spring barleys. However, its agronomic merit in winter barley is not clear. Previous studies indicate that this gene acts as a safeguard mechanism in winter barleys, promoting spikelet initiation under short days, and reducing vernalization requirement under long days (Casao et al., 2011b; Mulki et al., 2018). Also, *PPD-H2* seems to have an adaptive role, confirmed by its influence on key agronomic traits (Cuesta-Marcos et al., 2009; Mansour et al., 2018; Sharma et al., 2018; Monteagudo et al., 2019a). We have shown that one single functional *PPD-H2* allele in a winter barley hybrid does accelerate flowering under insufficient vernalization (provided a minimum vernalization threshold is supplied). Therefore, our data support the role of *PPD-H2* as a source of earliness, to promote timely growth in warm winters with incomplete vernalization, which can be included in the formulation of hybrids for areas with mild winters.

On the other hand, *VRN-H3* allelic variation also contributed to differences in the length of the late reproductive phase. The *vrn-H3c(1)* allele was associated with a short late reproductive phase independently of vernalization, showing partial dominance in the hybrids. This allele combines an early promoter with an early intron haplotype and has been associated with the earliest flowering in both, a landrace collection of predominantly winter barleys (SBCC) (Casas et al., 2011), and a cross of two spring cultivars (Casas et al., 2021). In contrast, the *vrn-H3d(1)* allele, characterized by a late promoter and early intron haplotype, seems to confer a long and constant duration of the reproductive phase, independently of vernalization. The late reproductive phase determines the potential number of grains; therefore, this allele could be a stable resource for breeders aiming at high-yielding varieties.

### Suggestions and Perspectives

This work shows the wide range of vernalization responses and flowering times that barley, and in particular hybrids, can display. From this information, the breeder can choose the earliness combination that best suits the conditions of each target environment. Here are some suggestions for allelic combinations that might work well, depending on the environment.

In climates where winters are long and cold, similar to our V8 treatment, and where we could expect that the vernalization needs will be completely satisfied, any of the tested hybrids would reach heading in a suitable date. However, to maximize yield, the breeder should choose a hybrid that matches the length of the LRP with the resource availability. In this sense, in environments prone to terminal stress, hybrids with a short LRP would have a better chance of escaping stress, which could be achieved with the *vrn-H3c(1)* allele or the *PPD-H2* allele. In contrast, in those environments where the end of the cycle benefits from optimal conditions, hybrids with a long LRP could enhance yield. In this scenario, the *vrn-H3d(1)* or *vrn-H3a(1)* allele could provide the effect we are looking for.

In those climates where winters are becoming warmer, comparable to our V4 treatment, where the cold needs might be compromised, the hybrid options that would flower on time are reduced. Hybrids carrying a dominant *PPD-H2* allele would be a suitable option when at least 4 weeks of cold were ensured. However, if the vernalization period decreased under 4 weeks, the delay in flowering time could negatively affect yield. A similar situation could be expected in hybrids with the *VRN-H1-6/vrn-H1* allelic combination. These could stand a certain decrease in the cold period duration, but not less than 4 weeks.

Under those circumstances, the safest option to ensure prompt flowering in the climate change scenario would be to use a hybrid with at least one *VRN-H1-4* allele or two *VRN-H1-6* alleles. Using these hybrid combinations should guarantee to maintain a suitable flowering even when the cold period is reduced under 4 weeks (V2 treatment).

In conclusion, although based on a small set of genotypes, we have demonstrated that hybrids can show a more nuanced response to insufficient vernalization than inbred lines. We also show that these phenotypic responses agree with the expression levels of main developmental genes. New options are proposed to manage time to flowering based on specific alleles and, particularly, the duration of developmental phases that build yield potential in hybrid barley. Our results highlight that hybrid combinations extend the available catalog of genetic responses to vernalization, which would be useful for adaptation to environmental conditions representing expected climate change trends.

## Data Availability Statement

The datasets presented in this study can be found in the Supplementary Tables 6–9 of the Supplementary Material section.

## Author Contributions

EI and AC conceived this work. FC and EI obtained the plant material. MF-C carried out the phenotyping and data collection. AC and MF-C performed the laboratory work. EI and MF-C performed the statistical analyses. MF-C, AC, and EI drafted the manuscript. All the authors read and approved the manuscript.

## Conflict of Interest

The authors declare that the research was conducted in the absence of any commercial or financial relationships that could be construed as a potential conflict of interest.

## Publisher’s Note

All claims expressed in this article are solely those of the authors and do not necessarily represent those of their affiliated organizations, or those of the publisher, the editors and the reviewers. Any product that may be evaluated in this article, or claim that may be made by its manufacturer, is not guaranteed or endorsed by the publisher.

## References

[B1] Ahmed T. A. Tsujimoto H. Sasakuma T. (2000). Identification of RFLP markers linked with heading date and its heterosis in hexaploid wheat. *Euphytica* 116 111–119. 10.1023/A:1004076826528

[B2] Al-Ashkar I. Alotaibi M. Refay Y. Ghazy A. Zakri A. Al-Doss A. (2020). Selection criteria for high-yielding and early-flowering bread wheat hybrids under heat stress. *PLoS One* 15:e0236351. 10.1371/journal.pone.0236351 32785293 PMC7423122

[B3] Alqudah A. M. Sharma R. Pasam R. K. Graner A. Kilian B. Schnurbusch T. (2014). Genetic Dissection of Photoperiod Response Based on GWAS of Pre-Anthesis Phase Duration in Spring Barley. *PLoS One* 9:e113120. 10.1371/journal.pone.0113120 25420105 PMC4242610

[B4] Barbosa-Neto J. F. Sorrells M. E. Cisar G. (1996). Prediction of heterosis in wheat using coefficient of parentage and RFLP-based estimates of genetic relationship. *Genome* 39 1142–1149. 18469962 10.1139/g96-144

[B5] Bernhard T. Friedt W. Voss-Fels K. P. Frisch M. Snowdon R. J. Wittkop B. (2017). Heterosis for biomass and grain yield facilitates breeding of productive dual-purpose winter barley hybrids. *Crop Sci.* 57 2405–2418. 10.2135/cropsci2016.10.0872 34798789

[B6] Bolaños J. Edmeades G. O. (1993). Eight cycles of selection for drought tolerance in lowland tropical maize. II. Responses in reproductive behavior. *F. Crop. Res.* 31 253–268. 10.1016/0378-4290(93)90065-U

[B7] Borghi B. Perenzin M. Nasht R. J. (1988). Agronomic and qualitative characteristics of ten bread wheat hybrids produced using a chemical hybridizing agent. *Euphytica* 39 185–194.

[B8] Campoli C. Shtaya M. Davis S. J. von Korff M. (2012). Expression conservation within the circadian clock of a monocot: natural variation at barley Ppd-H1 affects circadian expression of flowering time genes, but not clock orthologs. *BMC Plant Biol.* 12:97. 10.1186/1471-2229-12-97 22720803 PMC3478166

[B9] Campoli C. von Korff M. (2014). “Genetic Control of Reproductive Development in Temperate Cereals,” in *Advances in Botanical Research*, ed. Fornara F. (Cambridge: Academic Press), 131–158. 10.1016/B978-0-12-417162-6.00005-5

[B10] Casao M. C. Igartua E. Karsai I. Lasa J. M. Gracia M. P. Casas A. M. (2011a). Expression analysis of vernalization and day-length response genes in barley (Hordeum vulgare L.) indicates that VRNH2 is a repressor of PPDH2 (HvFT3) under long days. *J. Exp. Bot.* 62 1939–1949. 10.1093/jxb/erq382 21131547 PMC3060678

[B11] Casao M. C. Karsai I. Igartua E. Gracia M. P. Veisz O. Casas A. M. (2011b). Adaptation of barley to mild winters: A role for PPDH2. *BMC Plant Biol.* 11:164. 10.1186/1471-2229-11-164 22098798 PMC3226555

[B12] Casas A. M. Djemel A. Ciudad F. J. Yahiaoui S. Ponce L. J. Contreras-Moreira B. (2011). HvFT1 (VrnH3) drives latitudinal adaptation in Spanish barleys. *Theor. Appl. Genet.* 122 1293–1304. 10.1007/s00122-011-1531-x 21279626

[B13] Casas A. M. Gazulla C. R. Monteagudo A. Cantalapiedra C. P. Moralejo M. Pilar Gracia M. (2021). Candidate genes underlying QTL for flowering time and their interactions in a wide spring barley (Hordeum vulgare L.) cross. *Crop. J.* 9 862–872. 10.1016/J.CJ.2020.07.008

[B14] Cockram J. Jones H. Leigh F. J. O’Sullivan D. Powell W. Laurie D. A. (2007). Control of flowering time in temperate cereals: Genes, domestication, and sustainable productivity. *J. Exp. Bot.* 58 1231–1244. 10.1093/jxb/erm042 17420173

[B15] Contreras-Moreira B. Serrano-Notivoli R. Mohammed N. E. Cantalapiedra C. P. Beguería S. Casas A. M. (2019). Genetic association with high-resolution climate data reveals selection footprints in the genomes of barley landraces across the Iberian Peninsula. *Mol. Ecol.* 28 1994–2012. 10.1111/mec.15009 30614595 PMC6563438

[B16] Cooper M. Voss-Fels K. P. Messina C. D. Tang T. Hammer G. L. (2021). Tackling G × E × M interactions to close on-farm yield-gaps: creating novel pathways for crop improvement by predicting contributions of genetics and management to crop productivity. *Theor. Appl. Genet.* 134 1625–1644. 10.1007/s00122-021-03812-3 33738512 PMC8206060

[B17] Corbellini M. Perenzin M. Accerbi M. Vaccino P. Borghi B. (2002). Genetic diversity in bread wheat, as revealed by coefficient of parentage and molecular markers, and its relationship to hybrid performance. *Euphytica* 123 273–285. 10.1023/A:1014946018765

[B18] Craufurd P. Q. Wheeler T. R. (2009). Climate change and the flowering time of annual crops. *J. Exp. Bot.* 60 2529–2539. 10.1093/jxb/erp196 19505929

[B19] Cuesta-Marcos A. Casas A. M. Hayes P. M. Gracia M. P. Lasa J. M. Ciudad F. (2009). Yield QTL affected by heading date in Mediterranean grown barley. *Plant Breed.* 128 46–53. 10.1111/j.1439-0523.2008.01510.x

[B20] Deng W. Casao M. C. Wang P. Sato K. Hayes P. M. Finnegan E. J. (2015). Direct links between the vernalization response and other key traits of cereal crops. *Nat. Commun.* 6 5882. 10.1038/ncomms6882 25562483

[B21] Distelfeld A. Li C. Dubcovsky J. (2009). Regulation of flowering in temperate cereals. *Curr. Opin. Plant Biol.* 12 178–184. 10.1016/j.pbi.2008.12.010 19195924

[B22] Dreisigacker S. Melchinger A. E. Zhang P. Ammar K. Flachenecker C. Hoisington D. (2005). Hybrid performance and heterosis in spring bread wheat, and their relations to SSR-based genetic distances and coefficients of parentage. *Euphytica* 144 51–59. 10.1007/s10681-005-4053-2

[B23] Dubcovsky J. Chen C. Yan L. (2005). Molecular characterization of the allelic variation at the VRN-H2 vernalization locus in barley. *Mol. Breed.* 15 395–407. 10.1007/s11032-005-0084-6

[B24] Epskamp S. Cramer A. O. J. Waldorp L. J. Schmittmann V. D. Borsboom D. (2012). Qgraph: Network visualizations of relationships in psychometric data. *J. Stat. Softw.* 48 1–18. 10.18637/jss.v048.i04

[B25] Evans L. (1996). *Crop Evolution, Adaptation and Yield.* (Cambridge: Cambridge university press.)

[B26] Fatima Z. Ahmed M. Hussain M. Abbas G. Ul-Allah S. Ahmad S. (2020). The fingerprints of climate warming on cereal crops phenology and adaptation options. *Sci. Rep.* 10:18013. 10.1038/s41598-020-74740-3 33093541 PMC7581754

[B27] Faure S. Higgins J. Turner A. Laurie D. A. (2007). The *FLOWERING LOCUS T* -Like Gene Family in Barley (*Hordeum vulgare*). *Genetics* 176 599–609. 10.1534/genetics.106.069500 17339225 PMC1893030

[B28] Fernández-Calleja M. Casas A. M. Igartua E. (2021). Major flowering time genes of barley: allelic diversity, effects, and comparison with wheat. *Theor. Appl. Genet.* 134 1867–1897. 10.1007/s00122-021-03824-z 33969431 PMC8263424

[B29] Flohr B. M. Hunt J. R. Kirkegaard J. A. Evans J. R. Trevaskis B. Zwart A. (2018). Fast winter wheat phenology can stabilise flowering date and maximise grain yield in semi-arid Mediterranean and temperate environments. *F. Crop. Res.* 223 12–25. 10.1016/j.fcr.2018.03.021

[B30] Flood R. G. Halloran G. M. (1984). Basic Development Rate in Spring Wheat. *Agron. J.* 76 260–264. 10.2134/agronj1984.00021962007600020021x

[B31] Fu D. Szűcs P. Yan L. Helguera M. Skinner J. S. Von Zitzewitz J. (2005). Large deletions within the first intron in VRN-1 are associated with spring growth habit in barley and wheat. *Mol. Genet. Genomics* 273 54–65. 10.1007/s00438-004-1095-4 15690172

[B32] Gol L. Tomé F. von Korff M. (2017). Floral transitions in wheat and barley: interactions between photoperiod, abiotic stresses, and nutrient status. *J. Exp. Bot.* 68 1399–1410. 10.1093/jxb/erx055 28431134

[B33] González A. Martín I. Ayerbe L. (1999). Barley yield in water-stress conditions. The influence of precocity, osmotic adjustment and stomatal conductance. *F. Crop. Res.* 62 23–34. 10.1016/S0378-4290(99)00002-7

[B34] González F. G. Slafer G. A. Miralles D. J. (2002). Vernalization and photoperiod responses in wheat pre-flowering reproductive phases. *F. Crop. Res.* 74 183–195. 10.1016/S0378-4290(01)00210-6

[B35] Gouache D. Bogard M. Pegard M. Thepot S. Garcia C. Hourcade D. (2017). Bridging the gap between ideotype and genotype: Challenges and prospects for modelling as exemplified by the case of adapting wheat (Triticum aestivum L.) phenology to climate change in France. *F. Crop. Res.* 202 108–121. 10.1016/j.fcr.2015.12.012

[B36] Gracia M. P. Mansour E. Casas A. M. Lasa J. M. Medina B. Molina-Cano J. L. (2012). Progress in the Spanish National Barley Breeding Program. *Spanish J. Agric. Res.* 10 741–751. 10.5424/sjar/2012103-2613

[B37] Greenup A. G. Sasani S. Oliver S. N. Talbot M. J. Dennis E. S. Hemming M. N. (2010). ODDSOC2 is a MADS box floral repressor that is down-regulated by vernalization in temperate cereals. *Plant Physiol.* 153 1062–1073. 10.1104/pp.109.152488 20431086 PMC2899939

[B38] Griffiths F. E. W. Lyndon R. F. Bennett M. D. (1985). The Effects of Vernalization on the Growth of the Wheat Shoot Apex. *Ann. Bot.* 56 501–511. 10.1093/oxfordjournals.aob.a087035

[B39] Guerra D. Morcia C. Badeck F. Rizza F. Delbono S. Francia E. (2021). Extensive allele mining discovers novel genetic diversity in the loci controlling frost tolerance in barley. *Theor. Appl. Genet.* [Epub Online ahead of print]. 10.1007/S00122-021-03985-X 34757472 PMC8866391

[B40] Haas M. Himmelbach A. Mascher M. (2020). The contribution of cis- and trans-acting variants to gene regulation in wild and domesticated barley under cold stress and control conditions. *J. Exp. Bot.* 71 2573–2584. 10.1093/jxb/eraa036 31989179 PMC7210754

[B41] Hemming M. N. Fieg S. James Peacock W. Dennis E. S. Trevaskis B. (2009). Regions associated with repression of the barley (Hordeum vulgare) VERNALIZATION1 gene are not required for cold induction. *Mol. Genet. Genomics* 282 107–117. 10.1007/s00438-009-0449-3 19404679

[B42] Hemming M. N. Peacock W. J. Dennis E. S. Trevaskis B. (2008). Low-Temperature and Daylength Cues Are Integrated to Regulate FLOWERING LOCUS T in Barley. *Plant Physiol.* 147 355–366. 10.1104/pp.108.116418 18359843 PMC2330320

[B43] Hunt J. R. Lilley J. M. Trevaskis B. Flohr B. M. Peake A. Fletcher A. (2019). Early sowing systems can boost Australian wheat yields despite recent climate change. *Nat. Clim. Chang.* 9 244–247. 10.1038/s41558-019-0417-9

[B44] Karsai I. Szűcs P. Mészáros K. Filichkina T. Hayes P. M. Skinner J. S. (2005). The Vrn-H2 locus is a major determinant of flowering time in a facultative × winter growth habit barley (Hordeum vulgare L.) mapping population. *Theor. Appl. Genet.* 110 1458–1466. 10.1007/s00122-005-1979-7 15834697

[B45] Kassambara A. Mundt F. (2017). *Factoextra: Extract and Visualize the Results of Multivariate Data Analyses.* 337–354. Available online at: https://github.com/kassambara/factoextra/issues (Accessed on Dec 11, 2020)

[B46] Kikuchi R. Kawahigashi H. Ando T. Tonooka T. Handa H. (2009). Molecular and Functional Characterization of PEBP Genes in Barley Reveal the Diversification of Their Roles in Flowering. *Plant Physiol.* 149 1341–1353. 10.1104/pp.108.132134 19168644 PMC2649388

[B47] Kóti K. Karsai I. Sz P. Horváth C. Mészáros K. Kiss G. B. (2006). Validation of the two-gene epistatic model for vernalization response in a winter × spring barley cross. *Euphytica* 152 17–24. 10.1007/s10681-006-9170-z

[B48] Laurie D. A. Pratchett N. Snape J. W. Bezant J. H. (1995). RFLP mapping of five major genes and eight quantitative trait loci controlling flowering time in a winter × spring barley (*Hordeum vulgare* L.) cross. *Genome* 38 575–585. 10.1139/g95-074 18470191

[B49] Lê S. Josse J. Rennes A. Husson F. (2008). FactoMineR: An R Package for Multivariate Analysis. *JSS J. Stat. Softw.* 25 1–18. 10.18637/jss.v025.i01

[B50] Lenth R. Singmann H. Love J. Buerkner P. Herve M. (2018). Emmeans: Estimated marginal means, aka least-squares means. *R package version* .

[B51] Longin C. F. H. Gowda M. Mühleisen J. Ebmeyer E. Kazman E. Schachschneider R. (2013). Hybrid wheat: Quantitative genetic parameters and consequences for the design of breeding programs. *Theor. Appl. Genet.* 126 2791–2801. 10.1007/s00122-013-2172-z 23913277

[B52] Longin C. F. H. Mühleisen J. Maurer H. Zhang H. Gowda M. Reif J. C. (2012). Hybrid breeding in autogamous cereals. *Theor. Appl. Genet.* 125 1087–1096. 10.1007/s00122-012-1967-7 22918662

[B53] Loscos J. Igartua E. Contreras-Moreir B. Pilar Gracia M. Casas A. M. (2014). HvFT1 polymorphism and effect—survey of barley germplasm and expression analysis. *Front. Plant Sci.* 5:251. 10.3389/fpls.2014.00251 24936204 PMC4047512

[B54] Mansour E. Casas A. M. Gracia M. P. Molina-Cano J. L. Moralejo M. Cattivelli L. (2014). Quantitative trait loci for agronomic traits in an elite barley population for Mediterranean conditions. *Mol. Breed.* 33 249–265. 10.1007/s11032-013-9946-5

[B55] Mansour E. Moustafa E. S. A. Qabil N. Abdelsalam A. Wafa H. A. Kenawy A. (2018). Assessing different barley growth habits under Egyptian conditions for enhancing resilience to climate change. *F. Crop. Res.* 224 67–75. 10.1016/j.fcr.2018.04.016

[B56] Miralles D. J. Richards R. A. (2000). Responses of leaf and tiller emergence and primordium initiation in wheat and barley to interchanged photoperiod. *Ann. Bot.* 85 655–663. 10.1006/anbo.2000.1121

[B57] Monteagudo A. Casas A. M. Cantalapiedra C. P. Contreras-Moreira B. Gracia M. P. Igartua E. (2019a). Harnessing novel diversity from landraces to improve an elite barley variety. *Front. Plant Sci.* 10:434. 10.3389/fpls.2019.00434 31031782 PMC6470277

[B58] Monteagudo A. Igartua E. Contreras-Moreira B. Gracia M. P. Ramos J. Karsai I. (2019b). Fine-tuning of the flowering time control in winter barley: The importance of HvOS2 and HvVRN2 in non-inductive conditions. *BMC Plant Biol.* 19:113. 10.1186/s12870-019-1727-9 30909882 PMC6434887

[B59] Mühleisen J. Maurer H. P. Stiewe G. Bury P. Reif J. C. (2013). Hybrid Breeding in Barley. *Crop. Sci.* 53 819–824. 10.2135/CROPSCI2012.07.0411 34798789

[B60] Mühleisen J. Piepho H.-P. Maurer H. P. Longin C. F. H. Reif J. C. (2014). Yield stability of hybrids versus lines in wheat, barley, and triticale. *Theor. Appl. Genet.* 127 309–316. 10.1007/s00122-013-2219-1 24162154

[B61] Mulki M. A. Bi X. von Korff M. (2018). FLOWERING LOCUS T3 Controls Spikelet Initiation But Not Floral Development. *Plant Physiol.* 178 1170–1186. 10.1104/pp.18.00236 30213796 PMC6236595

[B62] Mulki M. A. von Korff M. (2016). CONSTANS Controls Floral Repression by Up-Regulating VERNALIZATION2 (VRN-H2) in Barley. *Plant Physiol.* 170 325–337. 10.1104/pp.15.01350 26556793 PMC4704585

[B63] Nitcher R. Distelfeld A. Tan C. Yan L. Dubcovsky J. (2013). Increased copy number at the HvFT1 locus is associated with accelerated flowering time in barley. *Mol. Genet. Genomics* 288 261–275. 10.1007/s00438-013-0746-8 23591592 PMC3664738

[B64] Ochagavía H. Prieto P. Savin R. Griffiths S. Slafer G. A. (2018). Dynamics of leaf and spikelet primordia initiation in wheat as affected by Ppd-1a alleles under field conditions. *J. Exp. Bot.* 69 2621–2631. 10.1093/jxb/ery104 29562296 PMC5920321

[B65] Oettler, Becker, and Hoppe. (2001). Heterosis for yield and other agronomic traits of winter triticale F1 and F2 hybrids. *Plant Breed.* 120 351–353. 10.1046/j.1439-0523.2001.00624.x

[B66] Olesen J. E. Trnka M. Kersebaum K. C. Skjelvåg A. O. Seguin B. Peltonen-Sainio P. (2010). Impacts and adaptation of European crop production systems to climate change. *Eur. J. Agron.* 34 96–112. 10.1016/j.eja.2010.11.003

[B67] Oliver S. N. Deng W. Casao M. C. Trevaskis B. (2013). Low temperatures induce rapid changes in chromatin state and transcript levels of the cereal VERNALIZATION1 gene. *J. Exp. Bot.* 64 2413–2422. 10.1093/jxb/ert095 23580755 PMC3654426

[B68] Oury F. Brabant P. Bérard P. Pluchard P. (2000). Predicting hybrid value in bread wheat: Biometric modelling based on a” top-cross” design. *Theor. Appl. Genet.* 100 96–104. 10.1007/PL00002905

[B69] Pagès J. (2002). Analyse factorielle multiple appliquée aux variables qualitatives et aux données mixtes. *Rev. Stat. appliquée* 50 5–37.

[B70] Porter J. R. Xie L. Challinor A. J. Chhetri Usa N. Garrett K. Aggarwal P. (2014). “Food security and food production systems,” in *Climate Change 2014: Impacts, Adaptation, and Vulnerability. Part A: Global and Sectoral Aspects. Contribution of Working Group II to the Fifth Assessment Report of the Intergovernmental Panel on Climate Change*, eds Field C. B. Barros V. R. Dokken D. J. Mach K. J. Mastrandrea M. D. Bilir T. E. (Cambridge, United Kingdom and New York, NY, USA: Cambridge University Press), 485–553.

[B71] R Core Team. (2013). *R: A language and environment for statistical computing.*(Vienna, Aus: R Foundation for Statistical Computing)

[B72] Roberts E. H. Summerfield R. J. Cooper J. P. Ellis R. H. (1988). Environmental Control of Flowering in Barley (Hordeum vulgare L.). I. Photoperiod Limits to Long-day Responses, Photoperiod-insensitive Phases and Effects of Low-temperature and Short-day Vernalization. *Ann. Bot.* 62 127–144. 10.1093/oxfordjournals.aob.a087644

[B73] Ruelens P. De Maagd R. A. Proost S. Theißen G. Geuten K. Kaufmann K. (2013). FLOWERING LOCUS C in monocots and the tandem origin of angiosperm-specific MADS-box genes. *Nat. Commun.* 4:2280. 10.1038/ncomms3280 23955420

[B74] Saadi S. Todorovic M. Tanasijevic L. Pereira L. S. Pizzigalli C. Lionello P. (2015). Climate change and Mediterranean agriculture: Impacts on winter wheat and tomato crop evapotranspiration, irrigation requirements and yield. *Agric. Water Manag.* 147 103–115. 10.1016/j.agwat.2014.05.008

[B75] Sasani S. Hemming M. N. Oliver S. N. Greenup A. Tavakkol-Afshari R. Mahfoozi S. (2009). The influence of vernalization and daylength on expression of flowering-time genes in the shoot apex and leaves of barley (*Hordeum vulgare*). *J. Exp. Bot.* 60 2169–2178. 10.1093/jxb/erp098 19357429 PMC2682508

[B76] Sharma R. Draicchio F. Bull H. Herzig P. Maurer A. Pillen K. (2018). Genome-wide association of yield traits in a nested association mapping population of barley reveals new gene diversity for future breeding. *J. Exp. Bot.* 69 3811–3822. 10.1093/jxb/ery178 29767798 PMC6054221

[B77] Sheehan H. Bentley A. (2021). Changing times: Opportunities for altering winter wheat phenology. *Plants People Planet* 3 113–123. 10.1002/ppp3.10163

[B78] Slafer G. A. Rawson H. M. (1994). Sensitivity of Wheat Phasic Development to Major Environmental Factors: a Re-Examination of Some Assumptions Made by Physiologists and Modellers. *Funct. Plant Biol.* 21 393–426. 10.1071/PP9940393

[B79] Stratonovitch P. Semenov M. A. (2015). Heat tolerance around flowering in wheat identified as a key trait for increased yield potential in Europe under climate change. *J. Exp. Bot.* 66 3599–3609. 10.1093/jxb/erv070 25750425 PMC4463804

[B80] Szűcs P. Skinner J. S. Karsai I. Cuesta-Marcos A. Haggard K. G. Corey A. E. (2007). Validation of the VRN-H2/VRN-H1 epistatic model in barley reveals that intron length variation in VRN-H1 may account for a continuum of vernalization sensitivity. *Mol. Genet. Genomics* 277 249–261. 10.1007/s00438-006-0195-8 17151889

[B81] Takahashi R. Yasuda S. (1971). “Genetics of earliness and growth habit in barley,” in *Barley genetics II. Proceeding 2nd International Barley Genetics Symposium*, ed. Nilan R. A. (Pullman: Washington State University Press), 388–408.

[B82] Tao F. Rötter R. P. Palosuo T. Díaz-Ambrona C. G. H. Mínguez M. I. Semenov M. A. (2017). Designing future barley ideotypes using a crop model ensemble. *Eur. J. Agron.* 82 144–162. 10.1016/j.eja.2016.10.012

[B83] Tondelli A. Francia E. Visioni A. Comadran J. Mastrangelo A. M. Akar T. (2014). QTLs for barley yield adaptation to Mediterranean environments in the “Nure” x “Tremois” biparental population. *Euphytica* 197 73–86. 10.1007/s10681-013-1053-5

[B84] Trevaskis B. Bagnall D. J. Ellis M. H. Peacock W. J. Dennis E. S. (2003). MADS box genes control vernalization-induced flowering in cereals. *Proc. Natl. Acad. Sci.U.S.A.* 100 13099–13104. 10.1073/pnas.1635053100 14557548 PMC240751

[B85] Trevaskis B. Hemming M. N. Dennis E. S. Peacock W. J. (2007). The molecular basis of vernalization-induced flowering in cereals. *Trends Plant Sci.* 12 352–357. 10.1016/j.tplants.2007.06.010 17629542

[B86] Trevaskis B. Hemming M. N. Peacock W. J. Dennis E. S. (2006). HvVRN2 responds to daylength, whereas HvVRN1 is regulated by vernalization and developmental status. *Plant Physiol.* 140 1397–1405. 10.1104/pp.105.073486 16500994 PMC1435809

[B87] Trnka M. Rötter R. P. Ruiz-Ramos M. Kersebaum K. C. Olesen J. E. Žalud Z. (2014). Adverse weather conditions for European wheat production will become more frequent with climate change. *Nat. Clim. Chang.* 4 637–643. 10.1038/nclimate2242

[B88] Turner A. Beales J. Faure S. Dunford R. P. Laurie D. A. (2005). The Pseudo-Response Regulator Ppd-H1 Provides Adaptation to Photoperiod in Barley. *Science* 310 1031–1034. 10.1126/science.1117619 16284181

[B89] von Zitzewitz J. Szűcs P. Dubcovsky J. Yan L. Francia E. Pecchioni N. (2005). Molecular and Structural Characterization of Barley Vernalization Genes. *Plant Mol. Biol.* 59 449–467. 10.1007/s11103-005-0351-2 16235110

[B90] Whitechurch E. M. Slafer G. A. Miralles D. J. (2007). Variability in the duration of stem elongation in wheat genotypes and sensitivity to photoperiod and vernalization. *J. Agron. Crop. Sci.* 193 131–137. 10.1111/j.1439-037X.2007.00259.x

[B91] Wiegmann M. Maurer A. Pham A. March T. J. Al-Abdallat A. Thomas W. T. B. (2019). Barley yield formation under abiotic stress depends on the interplay between flowering time genes and environmental cues. *Sci. Rep.* 9:6397. 10.1038/s41598-019-42673-1 31024028 PMC6484077

[B92] Yahiaoui S. Igartua E. Moralejo M. Ramsay L. Molina-Cano J. L. Ciudad F. J. (2008). Patterns of genetic and eco-geographical diversity in Spanish barleys. *Theor. Appl. Genet.* 116 271–282. 10.1007/s00122-007-0665-3 18026712

[B93] Yan L. Fu D. Li C. Blechl A. Tranquilli G. Bonafede M. (2006). The wheat and barley vernalization gene VRN3 is an orthologue of FT. *Proc. Natl. Acad. Sci.U.S.A.* 103 19581–19586. 10.1073/pnas.0607142103 17158798 PMC1748268

[B94] Yan L. Loukoianov A. Blechl A. Tranquilli G. Ramakrishna W. SanMiguel P. (2004). The Wheat VRN2 Gene Is a Flowering Repressor Down-Regulated by Vernalization. *Science* 303 1640–1644. 10.1126/science.1094305 15016992 PMC4737501

[B95] Yan L. Loukoianov A. Tranquilli G. Helguera M. Fahima T. Dubcovsky J. (2003). Positional cloning of the wheat vernalization gene VRN1. *Proc. Natl. Acad. Sci.U.S.A.* 100 6263–6268. 10.1073/pnas.0937399100 12730378 PMC156360

[B96] Yang C. Fraga H. van Ieperen W. Trindade H. Santos J. A. (2019). Effects of climate change and adaptation options on winter wheat yield under rainfed Mediterranean conditions in southern Portugal. *Clim. Change* 154 159–178. 10.1007/s10584-019-02419-4

[B97] Zadoks J. C. Chang T. T. Konzak C. F. (1974). A decimal code for the growth stages of cereals. *Weed Res.* 14 415–421. 10.1111/j.1365-3180.1974.tb01084.x

[B98] Zali A. A. Allard R. W. (1976). The effect of level of heterozygosity on the performance of hybrids between isogenic lines of barley. *Genetics* 84 765–775. 10.1093/genetics/84.4.765 17248734 PMC1213607

[B99] Zhao Y. Mette M. F. Gowda M. Longin C. F. H. Reif J. C. (2014). Bridging the gap between marker-assisted and genomic selection of heading time and plant height in hybrid wheat. *Heredity* 112 638–645. 10.1038/hdy.2014.1 24518889 PMC4023446

[B100] Zheng B. Chenu K. Fernanda Dreccer M. Chapman S. C. (2012). Breeding for the future: What are the potential impacts of future frost and heat events on sowing and flowering time requirements for Australian bread wheat (Triticum aestivium) varieties? *Glob. Chang. Biol.* 18 2899–2914. 10.1111/j.1365-2486.2012.02724.x 24501066

