# Supplementary material for: Hybrids Provide More Options for Fine-Tuning Flowering Time Responses of Winter Barley
Source: Front Plant Sci. 2022 Mar 22;13:827701. doi: 10.3389/fpls.2022.827701 (PMC9011329; doi:10.3389/fpls.2022.827701)
Supplement: Supplementary file 1 [file Data_Sheet_1.pdf]

Supplementary Material

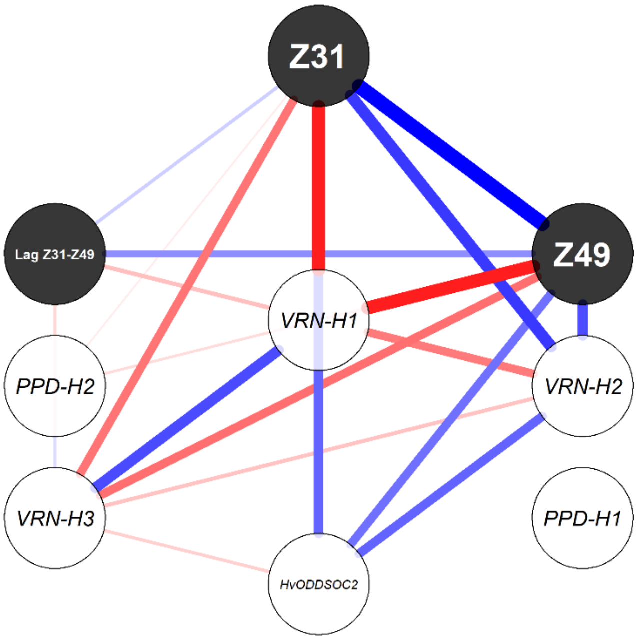

Supplementary Figure 1. Correlation network of developmental phases and flowering time regulators gene expression (averaged across two sampling times per genotype by treatment combination, 33 data points). Red and blue lines represent negative and positive correlations, respectively. Line width is proportional to the strength of the correlation. Only significant (P<0.05) correlations are shown. Developmental phases depicted in black: days to first node appearance (Z31), days to awn tipping (Z49), and late reproductive phase (Lag Z31-Z49). Flowering time genes expression depicted in white: *VRN-H1*, *VRN-H2*, *PPD-H1*, *HvODDSOC2*, *VRN-H3*, and *PPD-H2*.

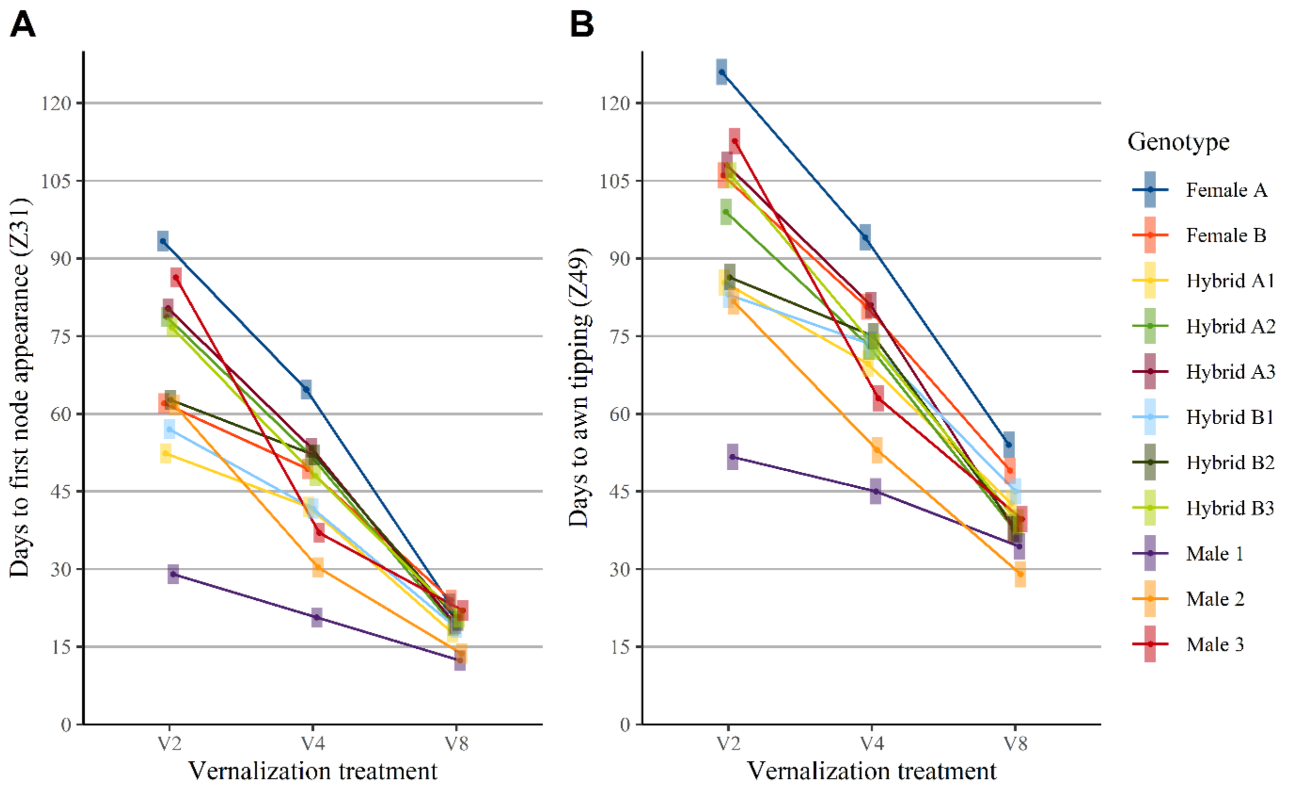

Supplementary Figure 2. Vernalization sensitivity of days to first node appearance (A) and days to awn tipping (B). Dots represent means for each genotype and vernalization treatment (V2: 2 weeks of vernalization, V4: 4 weeks of vernalization, V8: 8 weeks of vernalization). Each colour line connects the treatment means from one genotype. Error bars are 95% confidence intervals.

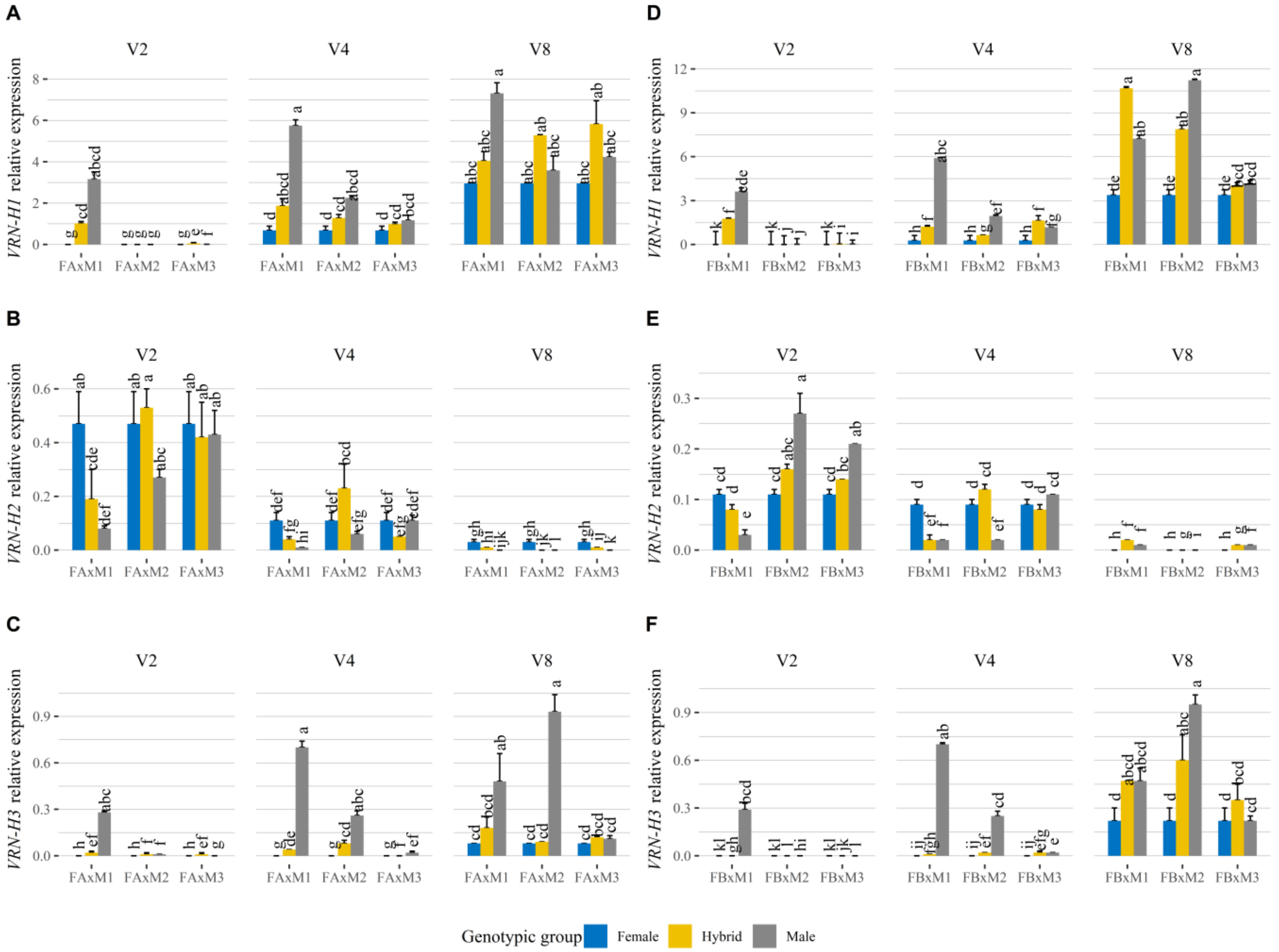

Supplementary Figure 3. Relative expression levels of *VRN-H1* (A, D), *VRN-H2* (B, E) and *VRN-H3* (C, F) assayed by qRT-PCR in triads of barley genotypes (Female, Hybrid, Male) grown under 16 h light, in response to different vernalization treatments (V2: 2 weeks of vernalization, V4: 4 weeks of vernalization, V8: 8 weeks of vernalization; 4-8 °C, 8 h light). Plots A, B and C correspond to female A crosses (Batch A). Plots D, E and F correspond to female B crosses (Batch B). Each plot is divided in three facets, each of them containing gene expression assayed for one vernalization treatment, and the three triads of genotypes composed of one female parent in blue, the male parent in grey, and the hybrid in yellow. The triads are represented as abbreviations of the crosses between the parents, e.g., FAxM1: Female A x Male 1. The results shown are normalized to the level of the housekeeping gene *Actin* for each genotype and treatment. Samples were taken from plants after 17 d of growth under each treatment. Mean of 3 biological replicates. Error bars represent the SEM. For each gene and batch, bars with a different letter are significantly different at $P < 0.05$, according to ANOVA that included genotypes and all treatments.

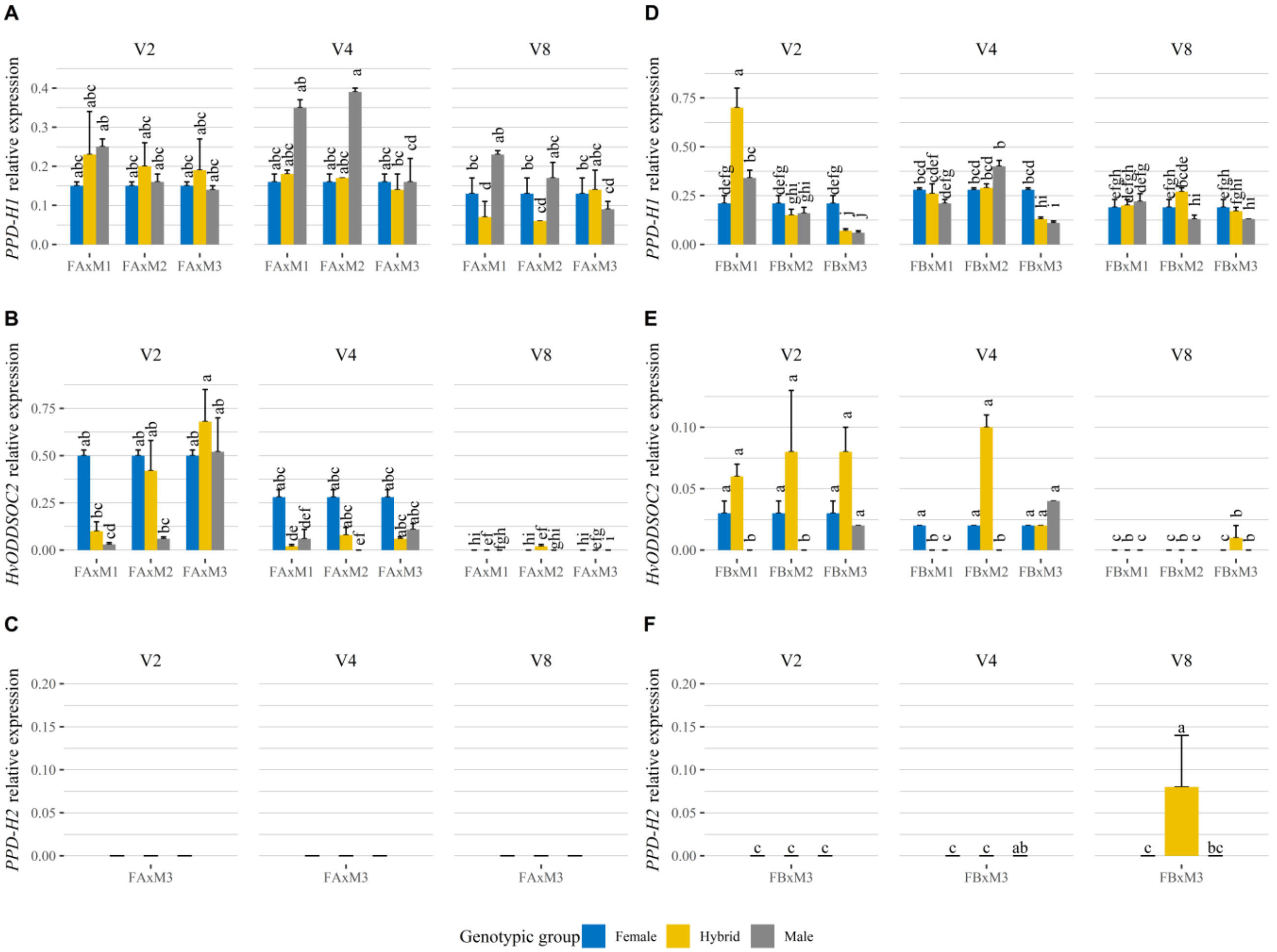

Supplementary Figure 4. Relative expression levels of *PPD-H1* (A, D), *HvODDSOC2* (B, E) and *PPD-H2* (C, F) assayed by qRT-PCR in triads of barley genotypes (Female, Hybrid, Male) grown under 16 h light, in response to different vernalization treatments (V2: 2 weeks of vernalization, V4: 4 weeks of vernalization, V8: 8 weeks of vernalization; 4-8 °C, 8 h light). Plots A, B and C correspond to female A crosses (Batch A). Plots D, E and F correspond to female B crosses (Batch B). Each plot is divided in three facets, each of them containing gene expression assayed for one vernalization treatment, and the three triads of genotypes composed of one female parent in blue, the male parent in grey, and the hybrid in yellow. The triads are represented as abbreviations of the crosses between the parents, e.g., FBxM1: Female B x Male 1. The results shown are normalized to the level of the housekeeping gene *Actin* for each genotype and treatment. Samples were taken from plants after 35 d of growth under each treatment. Mean of 3 biological replicates. Error bars represent the SEM. For each gene and batch, bars with a different letter are significantly different at $P < 0.05$, according to ANOVA that included genotypes and all treatments.

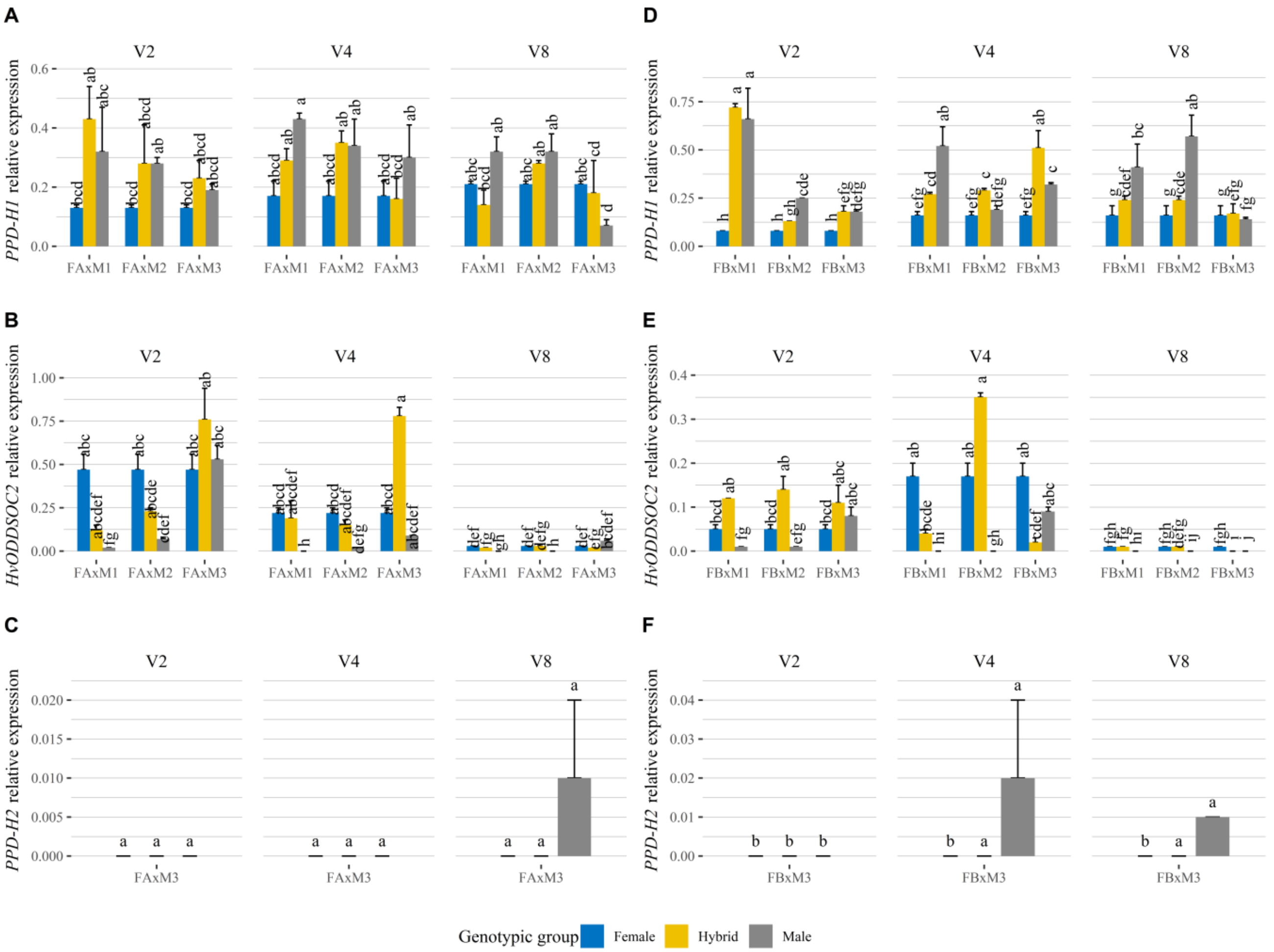

Supplementary Figure 5. Relative expression levels of *PPD-H1* (A, D), *HvODDSOC2* (B, E) and *PPD-H2* (C, F) assayed by qRT-PCR in triads of barley genotypes (Female, Hybrid, Male) grown under 16 h light, in response to different vernalization treatments (V2: 2 weeks of vernalization, V4: 4 weeks of vernalization, V8: 8 weeks of vernalization; 4-8 °C, 8 h light). Plots A, B and C correspond to female A crosses (Batch A). Plots D, E and F correspond to female B crosses (Batch B). Each plot is divided in three facets, each of them containing gene expression assayed for one vernalization treatment, and the three triads of genotypes composed of one female parent in blue, the male parent in grey, and the hybrid in yellow. The triads are represented as abbreviations of the crosses between the parents, e.g., FBxM1: Female B x Male 1. The results shown are normalized to the level of the housekeeping gene *Actin* for each genotype and treatment. Samples were taken from plants after 17 d of growth under each treatment. Mean of 3 biological replicates. Error bars represent the SEM. For each gene and batch, bars with a different letter are significantly different at $P < 0.05$, according to ANOVA that included genotypes and all treatments.

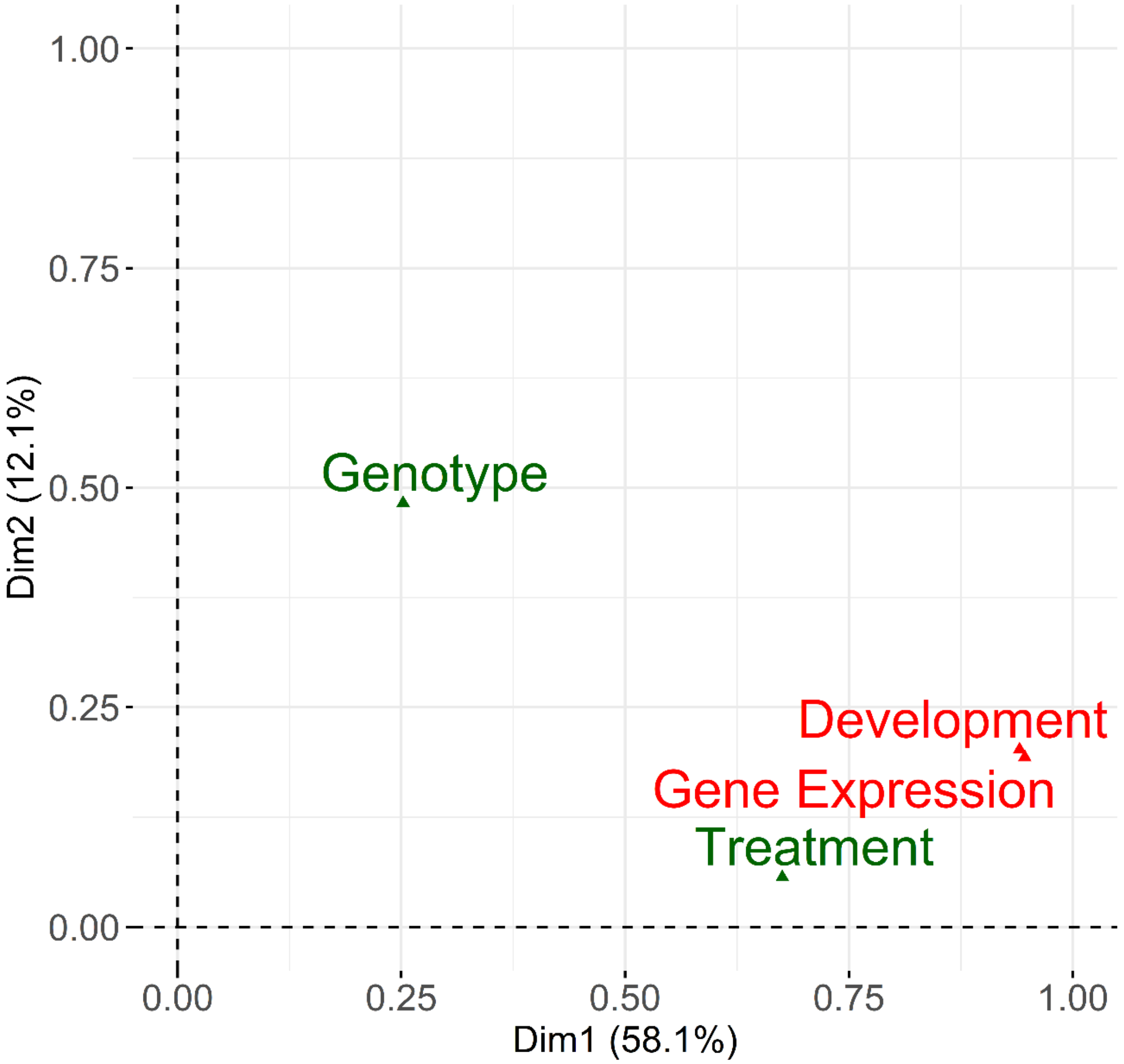

Supplementary Figure 6. Multiple factorial analysis (MFA), plot of variable group correlation with axes. The plot represents the correlation between groups of variables and axes. *Development* and *Gene Expression* (red) are active groups based on quantitative variables used to define the dimensions of the MFA. *Genotype* and *treatment* (green) are supplementary groups based on categorical variables specifying the genotype identity of each individual and the vernalization treatment to which they were subjected. Triangles represent the correlation of groups with the axes.

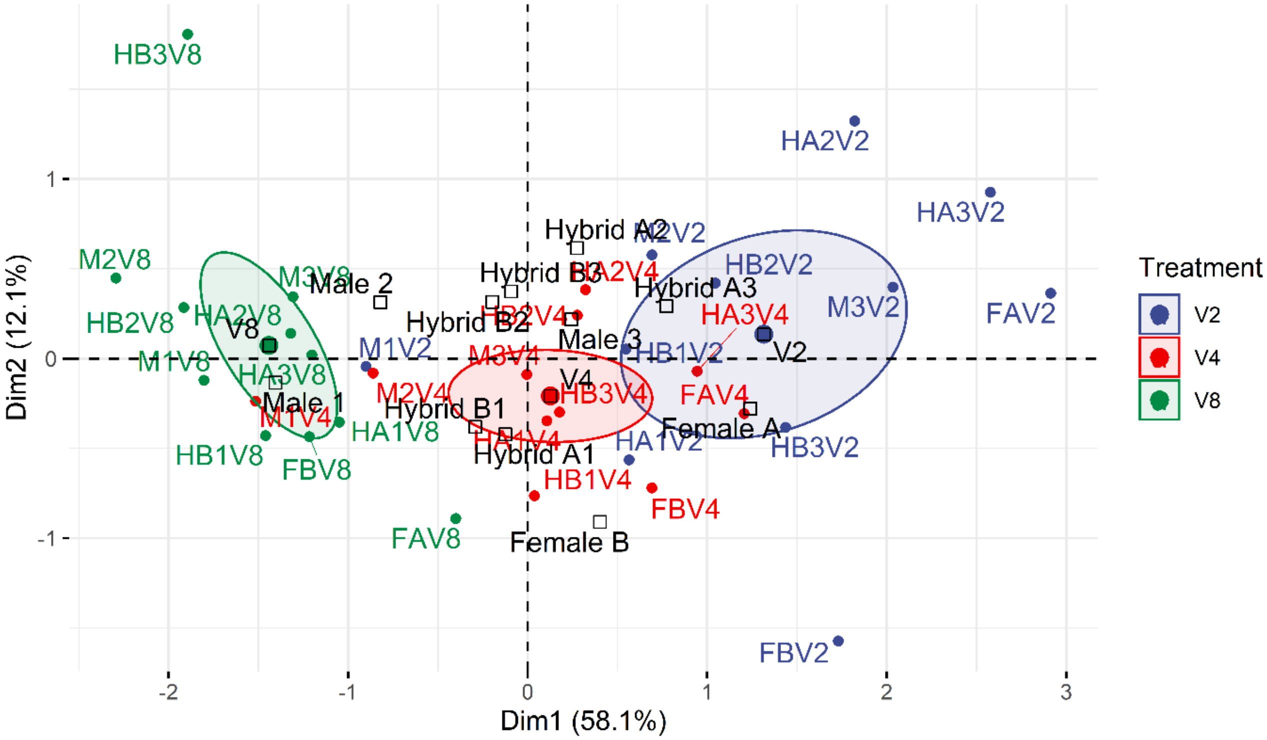

Supplementary Figure 7. Multiple factorial analysis (MFA). Individuals grouped by vernalization treatment. This plot exhibits the position of individuals in the MFA by the vernalization treatment. Dots represent individuals (e.g., FAV2: genotype Female A in 2-week vernalization treatment). Squares represent group mean points for categorial variables. Confidence interval ellipses around each vernalization treatment were added. V2: 2 weeks of cold treatment; V4: 4 weeks of cold treatment; V8: 8 weeks of cold treatment.

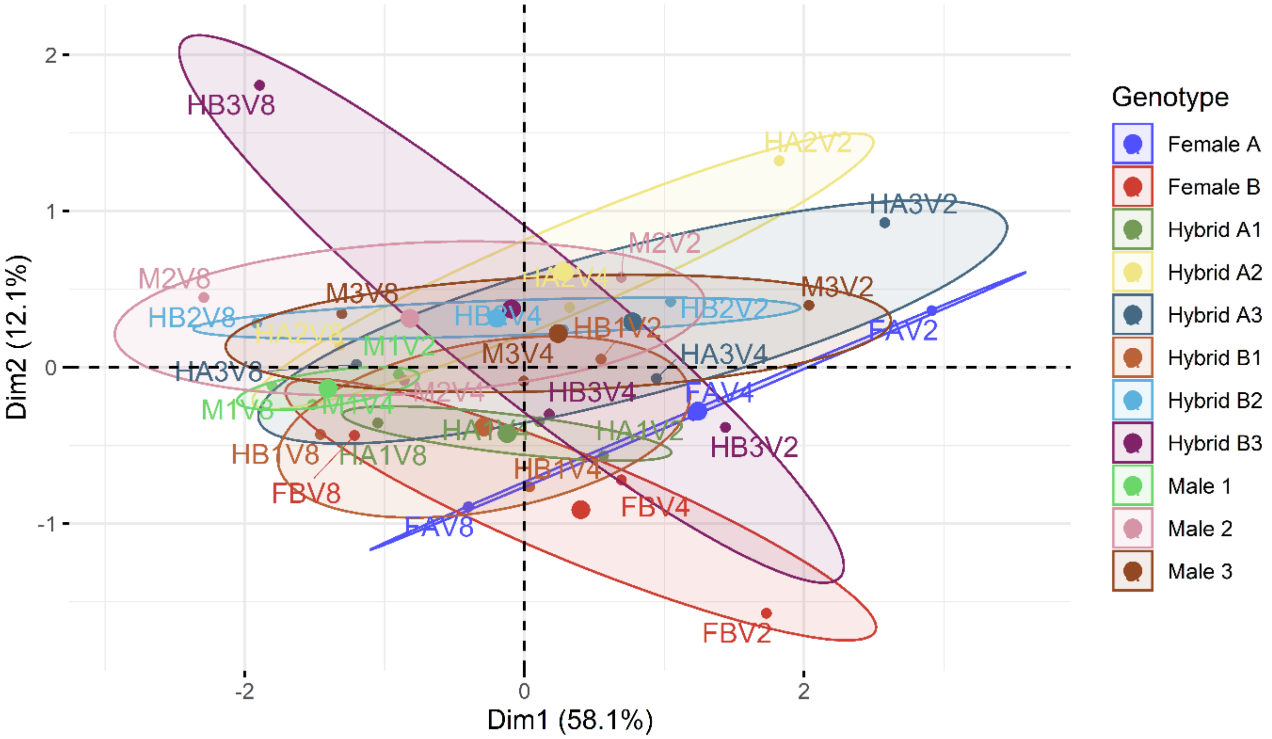

Supplementary Figure 8. Multiple factorial analysis (MFA). Individuals grouped by genotype. This plot exhibits the position of individuals in the MFA by the genotype variation. Dots represent individuals (e.g., HB3V8: genotype Hybrid B3 in 8-week vernalization treatment). Confidence interval ellipses around each genotype were added.

Supplementary Table 1. Primer sequences for gene expression assay.

Gene	Primer sequence (5'-3')	Reference
VRN-H1[a]	Forward: TGAAGCTCAGAAATGGATTCG<br>Reverse: TATGAGCGCTACTCTTATGC	Trevaskis et al. (2006)
VRN-H2[a]	Forward: GAGCCACCATCGTGCCATTC<br>Reverse: GCCGCTTCTTCCTCTTCTC	Trevaskis et al. (2006)
VRN-H3[a]	Forward: ATCTCCACTGGTTGGTGACAGA<br>Reverse: TTGTAGAGCTCGGCAAAGTCC	Yan et al. (2006)
PPD-H1[a]	Forward: CAAATCAAAGAGCGGCGATC<br>Reverse: TCTGACTTGGGATGGTTCACA	Hemming et al. (2008)
HvODDSOC2[a]	Forward: CAATGCTGATGACTCAGATGCT<br>Reverse: CGCTATTTCGTTGCGCCAAT	Greenup et al. (2010)
PPD-H2[b]	Forward: GGTTGTGGCTCATGTTATGC<br>Reverse: CTACTCCCCTTGAGAACTTTC	F: Kikuchi et al. (2009)<br>R: Faure et al. (2007)
Actin[a]	Forward: GCCGTGCTTTCCCTCTATG<br>Reverse: GCTTCTCCTTGATGTCCCTTA	Trevaskis et al. (2006)

[a]For these genes, each reaction contained 5 µl of PowerUp SYBR Green Master Mix (Applied Biosystems), 0.5 µM of each primer and 250 ng of cDNA in a volume of 10 µl. Reactions were run with the following conditions: 2 min at 50°C, 2 min at 95°C, 44 cycles of 15 s at 95°C and 1 min at 60°C, followed by a melting curve program (60-95°C) implying temperature increases of 1°C each minute.

[b]For this gene, each reaction contained 5 µl of PowerUp SYBR Green Master Mix (Applied Biosystems), 0.5 µM of each primer and 250 ng of cDNA in a volume of 10 µl. Reactions were run with the following conditions: 2 min at 50°C, 2 min at 95°C, 44 cycles of 15 s at 95°C, 15 s at 60°C and 45 s at 72°C, and a melting curve program (60-95°C) of 1°C of temperature increment for each minute.

Supplementary Table 2. Effects of genotype, repetition, treatment, genotype by treatment interaction, and contrasts on developmental variables. The values under each variable's heading correspond to mean squares.

**Source of variation**	**Df**	**Z31**	**Z49**	**Lag Z31-Z49**	**Sensitivity Z31**	**Sensitivity Z49**	**Sensitivity Lag Z31-Z49**
Genotype	10	976.0***	1373.2***	183.2***	375.2***	440.2***	48.4***
F_1 *vs.* Parents	1	241.0***	64.0***	56.6**	7.2	0.2	5.0
within Parents	4	2008.0***	3246.7***	314.7***	686.5***	764.8***	81.5***
Females *vs.* Males	1	3499.2***	8636.0***	1140.8***	217.7***	510.0***	61.2**
within Females	1	1058.0***	734.7***	29.4*	768.2***	168.7***	216.7***
within Males	2	1737.4***	1808.1***	44.3**	880.2***	1190.2***	24.0
within Hybrids	5	297.3***	136.2***	103.3***	199.7***	268.6***	30.6**
F_1 (Fem A) *vs.* F_1 (Fem B)	1	50.1***	42.7**	0.3	83.9***	103.4***	1.0
within F_1 (Fem A)	2	518.5***	211.8***	160.6***	330.1***	297.2***	27.6*
within F_1 (Fem B)	2	199.7***	107.4***	97.4***	127.2***	322.7***	48.4**
Repetition	2	1.0	11.0	10.9	5.0	3.7	10.0
Treatment	2	19126.4***	24628.5***	370.7***	133.9***	661.8***	200.4***
Genotype*Treatment	20	248.2***	267.5***	33.1***	363.9***	284.4***	53.7***
Residuals	64	2.8	4.9	6.6	2.8	4.6	7.5

Df, degrees of freedom; P < 0.1 * P < 0.05 ** P <0.01 *** P <0.001.

Supplementary Table 3. Means and 95% confidence intervals of developmental phases for each genotypic set by treatment combination. For each developmental variable and treatment, group means with a different letter are significantly different at $P < 0.05$ according to the contrasts performed for the overall ANOVA.

**Genotypic set**	**Treatment**	**Z31±CI**	**Z49±CI**	**LagZ31-Z49±CI**
Female	V2	77.7 ± 8.7 ^{a}	116.0 ± 8.5 ^{a}	38.3 ± 3.4 ^{a}
Hybrid	V2	67.9 ± 5.1 ^{b}	94.6 ± 4.9 ^{b}	26.7 ± 2.0 ^{b}
Male	V2	59.0 ± 7.1 ^{c}	82.0 ± 6.9 ^{c}	23.0 ± 2.8 ^{c}
Female	V4	57.0 ± 8.7 ^{a}	87.3 ± 8.5 ^{a}	30.3 ± 3.4 ^{a}
Hybrid	V4	48.2 ± 5.0 ^{b}	74.2 ± 4.9 ^{b}	26.0 ± 2.0 ^{b}
Male	V4	29.3 ± 7.2 ^{c}	53.7 ± 7.0 ^{c}	24.3 ± 2.8 ^{b}
Female	V8	23.7 ± 8.7 ^{a}	51.5 ± 8.5 ^{a}	27.8 ± 3.4 ^{a}
Hybrid	V8	19.2 ± 5.1 ^{b}	40.0 ± 4.9 ^{b}	20.8 ± 2.0 ^{b}
Male	V8	16.0 ± 7.1 ^{c}	34.3 ± 7.0 ^{c}	18.3 ± 2.8 ^{b}

CI, 95% confidence interval.

Supplementary Table 4. Effects of genotype, repetition, treatment, sampling time, factorial interactions, and contrasts on gene expression for Batch A. The values under each variable's heading correspond to mean squares.

Source of variation	Df	dCt*VRN-H1*	dCt*VRN-H2*	dCt*VRN-H3*	dCt*PPD-H1*	dCt*PPD-H2*	dCt*HvODDSOC2*
Genotype	6	86.6***	48.6***	122.6***	4.9***	3.4	74.3***
F$_1$ *vs.* Parents	1	2.9	31.6***	5.7*	4.2	2.6	105.0***
F$_1$ *vs.* Females	1	105.0***	29.8***	170.5***	0.0	0.9	1.4
F$_1$ *vs.* Males	1	7.4	91.8***	83.5***	6.3*	3.4	177.6***
within Parents	3	122.6***	83.4***	215.8***	7.7***	0.8	97.7***
Females *vs.* Males	1	148.1***	149.6***	381.1***	2.9	0.8	112.2***
within Males	2	109.9***	50.3***	133.2***	10.1***		90.6***
within Hybrids	2	74.4***	4.9**	41.1***	1.0		23.9*
Repetition	2	2.0	2.3	1.4	0.2	5.7	4.4
Treatment	2	866.0***	506.4***	271.1***	6.0**	5.7	586.3***
Genotype*Treatment	12	48.8***	18.0***	22.3***	1.3	23.7	12.3*
Sampling Time (ST)	1	142.8***	44.4***	162.1***	8.8**	0.8	249.7***
Genotype*Sampling Time	6	8.9**	2.9**	12.1***	1.2	24.7	7.3
Treatment*Sampling Time	2	112.9***	12.9***	5.0*	0.6	8.5	117.1***
Genotype*Treatment*ST	12	9.1***	2.1*	5.2***	1.1	30.0	10.9*
Residuals	82	2.4	0.9	1.4	1.1	341.1	5.4

Df, degrees of freedom; $P < 0.1$ * $P < 0.05$ ** $P < 0.01$ *** $P < 0.001$.

Supplementary Table 5. Effects of genotype, repetition, treatment, sampling time, factorial interactions, and contrasts on gene expression for Batch B. The values under each variable's heading correspond to mean squares.

Source of variation	Df	dCt*VRN-H1*	dCt*VRN-H2*	dCt*VRN-H3*	dCt*PPD-H1*	dCt*PPD-H2*	dCt*HvODDSOC2*
Genotype	6	46.9***	12.8***	87.8***	5.3***	156.2***	117.2***
F_1 *vs.* Parents	1	0.0	10.1***	41.9***	0.8*	23.9	303.1***
F_1 *vs.* Females	1	52.7***	0.1	23.6***	3.5***	161.9**	7.2
F_1 *vs.* Males	1	10.1***	16.7***	107.3***	0.1	18.2	418.2***
within Parents	3	68.6***	22.1***	150.3***	6.8***	288.6***	114.3***
Females *vs.* Males	1	90.3***	9.8***	148.3***	2.9***	288.5***	138.9***
within Males	2	57.8***	28.2***	151.3***	8.8***	0.0	102.1***
within Hybrids	2	37.7***	0.3	17.0***	5.3***	0.0	28.5***
Repetition	2	0.0	0.3	0.8	0.0	19.6	17.6**
Treatment	2	479.5***	204.6***	717.1***	1.9***	155.3***	461.6***
Genotype*Treatment	12	22.9***	10.7***	25.5***	2.0***	58.6**	18.8***
SamplingTime (ST)	1	51.5***	0.6	337.8***	6.3***	28.7	124.7***
Genotype*SamplingTime	6	4.7***	3.0***	3.5**	2.6***	23.4	15.0***
Treatment*SamplingTime	2	20.8***	25.3***	6.2**	0.0	18.9	8.1
Genotype*Treatment*ST	12	3.0***	3.2***	3.0***	1.3***	30.5	7.9**
Residuals	82	0.3	0.2	0.9	0.2	14.7	2.8

Df, degrees of freedom; P < 0.1 * P < 0.05 ** P <0.01 *** P <0.001.

Supplementary Table 6. Means and standard deviation of developmental variables for each genotype by treatment combination.

Genotype	Treatment	Z31±SD	Z49±SD	LagZ31-Z49±SD
Female A	V2	93.33±0.58	126.00±2.00	32.67±2.08
Female B	V2	62.00±2.65	106.00±1.00	44.00±1.73
Hybrid A1	V2	52.33±0.58	85.33±2.08	33.00±1.73
Hybrid A2	V2	78.67±3.79	99.00±3.61	20.33±1.53
Hybrid A3	V2	80.33±1.53	108.00±1.00	27.67±0.58
Hybrid B1	V2	57.00±1.00	83.00±2.65	26.00±1.73
Hybrid B2	V2	62.67±0.58	86.33±0.58	23.67±1.15
Hybrid B3	V2	76.67±0.58	106.00±5.00	29.33±5.51
Male 1	V2	29.00±1.00	51.67±2.08	22.67±1.53
Male 2	V2	61.67±0.58	81.67±2.52	20.00±2.00
Male 3	V2	86.33±0.58	112.67±0.58	26.33±0.58
Female A	V4	64.67±4.04	94.00±0.00	29.33±4.04
Female B	V4	49.33±1.15	80.67±3.79	31.33±4.93
Hybrid A1	V4	42.00±3.00	69.67±3.06	27.67±3.51
Hybrid A2	V4	52.00±1.00	73.00±1.00	21.00±1.73
Hybrid A3	V4	53.33±0.58	81.00±1.00	27.67±0.58
Hybrid B1	V4	41.67±0.58	73.33±0.58	31.67±1.15
Hybrid B2	V4	52.00±1.00	75.00±2.00	23.00±1.73
Hybrid B3	V4	48.00±3.46	73.00±0.00	25.00±3.46
Male 1	V4	20.67±1.53	45.00±1.00	24.33±0.58
Male 2	V4	30.33±3.21	53.00±0.00	22.67±3.21
Male 3	V4	37.00±1.73	63.00±0.00	26.00±1.73
Female A	V8	23.33±1.53	54.00±1.00	30.67±0.58
Female B	V8	24.00±1.00	49.00±1.73	25.00±2.65
Hybrid A1	V8	17.67±0.58	42.67±4.16	25.00±3.61
Hybrid A2	V8	19.00±0.00	38.00±3.46	19.00±3.46
Hybrid A3	V8	19.33±0.58	37.67±4.04	18.33±4.51
Hybrid B1	V8	18.67±0.58	45.00±2.00	26.33±2.52
Hybrid B2	V8	20.00±1.00	37.67±2.52	17.67±3.21
Hybrid B3	V8	20.67±1.15	39.00±2.00	18.33±2.31
Male 1	V8	12.33±0.58	34.33±0.58	22.00±1.00
Male 2	V8	13.67±0.58	29.00±0.00	15.33±0.58
Male 3	V8	22.00±0.00	39.67±2.08	17.67±2.08

SD, standard deviation.

Supplementary Table 7. Means and standard deviation of vernalization sensitivity of developmental phases for each genotype by treatment combination.

Genotype	Treatment	Sensitivity Z31±SD	Sensitivity Z49±SD	Sensitivity Lag Z31-Z49±SD
Female A	V4-V2	-28.67±4.04	-32.00±0.00	-3.33±4.04
Female B	V4-V2	-12.67±1.15	-25.33±3.79	-12.67±4.93
Hybrid A1	V4-V2	-10.33±3.00	-15.67±3.06	-5.33±3.51
Hybrid A2	V4-V2	-26.67±1.00	-26.00±1.00	0.67±1.73
Hybrid A3	V4-V2	-27.00±0.58	-27.00±1.00	0.00±0.58
Hybrid B1	V4-V2	-15.33±0.58	-9.67±0.58	5.67±1.15
Hybrid B2	V4-V2	-10.67±1.00	-11.33±2.00	-0.67±1.73
Hybrid B3	V4-V2	-28.67±3.46	-33.00±0.00	-4.33±3.46
Male 1	V4-V2	-8.33±1.53	-6.67±1.00	1.67±0.58
Male 2	V4-V2	-31.33±3.21	-28.67±0.00	2.67±3.21
Male 3	V4-V2	-49.33±1.73	-49.67±0.00	-0.33±1.73
Female A	V8-V4	-41.33±1.53	-40.00±1.00	1.33±0.58
Female B	V8-V4	-25.33±1.00	-31.67±1.73	-6.33±2.65
Hybrid A1	V8-V4	-24.33±0.58	-27.00±4.16	-2.67±3.61
Hybrid A2	V8-V4	-33.00±0.00	-35.00±3.46	-2.00±3.46
Hybrid A3	V8-V4	-34.00±0.58	-43.33±4.04	-9.33±4.51
Hybrid B1	V8-V4	-23.00±0.58	-28.33±2.00	-5.33±2.52
Hybrid B2	V8-V4	-32.00±1.00	-37.33±2.52	-5.33±3.21
Hybrid B3	V8-V4	-27.33±1.15	-34.00±2.00	-6.67±2.31
Male 1	V8-V4	-8.33±0.58	-10.67±0.58	-2.33±1.00
Male 2	V8-V4	-16.67±0.58	-24.00±0.00	-7.33±0.58
Male 3	V8-V4	-15.00±0.00	-23.33±2.08	-8.33±2.08

SD, standard deviation.

Supplementary Table 8. Means and standard error of relative gene expression for each genotype by treatment combination of Batch A.

Genotype	Treatment	Sampling Time	*VRN-H1*±SE	*VRN-H2*±SE	*VRN-H3*±SE	*PPD-H1*±SE	*PPD-H2*±SE	*HvODDSOC2*±SE
Female A	V2	17	0.000±0.000	0.4705±0.1154	0.0000±0.0000	0.125±0.007	0.0000±0.0000	0.4699±0.0898
Hybrid A1	V2	17	1.025±0.089	0.1861±0.1116	0.0152±0.0053	0.430±0.114		0.1174±0.0284
Hybrid A2	V2	17	0.000±0.000	0.5337±0.0680	0.0108±0.0073	0.279±0.133		0.2285±0.0177
Hybrid A3	V2	17	0.046±0.041	0.4237±0.1268	0.0142±0.0067	0.230±0.063	0.0000±0.0000	0.7640±0.1806
Male 1	V2	17	3.160±0.347	0.0772±0.0069	0.2816±0.0068	0.318±0.146		0.0161±0.0006
Male 2	V2	17	0.000±0.000	0.2735±0.0324	0.0070±0.0027	0.284±0.024		0.0703±0.0098
Male 3	V2	17	0.008±0.004	0.4323±0.0890	0.0003±0.0001	0.185±0.020	0.0000±0.0000	0.5344±0.0835
Female A	V4	17	0.682±0.199	0.1137±0.0338	0.0006±0.0003	0.165±0.050	0.0000±0.0000	0.2236±0.0295
Hybrid A1	V4	17	1.872±0.351	0.0437±0.0077	0.0362±0.0023	0.289±0.039		0.1929±0.0957
Hybrid A2	V4	17	1.283±0.170	0.2280±0.0907	0.0836±0.0190	0.352±0.041		0.1495±0.0334
Hybrid A3	V4	17	0.982±0.101	0.0524±0.0033	0.0048±0.0025	0.157±0.069	0.0019±0.0018	0.7823±0.0505
Male 1	V4	17	5.751±0.279	0.0115±0.0014	0.7049±0.0408	0.434±0.021		0.0011±0.0001
Male 2	V4	17	2.250±0.107	0.0588±0.0075	0.2588±0.0334	0.336±0.091		0.0224±0.0015
Male 3	V4	17	1.174±0.239	0.1051±0.0184	0.0217±0.0057	0.300±0.114	0.0000±0.0000	0.0877±0.0024
Female A	V8	17	2.954±0.037	0.0325±0.0076	0.0785±0.0025	0.206±0.013	0.0000±0.0000	0.0322±0.0026
Hybrid A1	V8	17	4.039±0.455	0.0136±0.0049	0.1765±0.0707	0.139±0.050		0.0160±0.0023
Hybrid A2	V8	17	5.281±0.033	0.0042±0.0013	0.0852±0.0021	0.277±0.008		0.0282±0.0055
Hybrid A3	V8	17	5.825±1.134	0.0081±0.0022	0.1222±0.0079	0.183±0.109	0.0000±0.0000	0.0233±0.0042
Male 1	V8	17	7.312±0.505	0.0044±0.0004	0.4832±0.1755	0.321±0.054		0.0030±0.0004
Male 2	V8	17	3.576±0.697	0.0000±0.0000	0.9338±0.1077	0.323±0.058		0.0007±0.0001
Male 3	V8	17	4.228±0.235	0.0020±0.0003	0.1132±0.0230	0.066±0.016	0.0120±0.0120	0.0592±0.0087
Female A	V2	35	0.023±0.018	0.4869±0.0402	0.0011±0.0001	0.152±0.008	0.0000±0.0000	0.4979±0.0297
Hybrid A1	V2	35	2.569±0.335	0.0684±0.0169	1.5313±0.4782	0.234±0.110		0.1043±0.0545
Hybrid A2	V2	35	0.059±0.025	0.4978±0.0605	0.0116±0.0034	0.203±0.057		0.4203±0.1559
Hybrid A3	V2	35	0.154±0.048	0.4706±0.1408	0.0113±0.0062	0.191±0.078	0.0000±0.0000	0.6826±0.1664
Male 1	V2	35	4.054±0.197	0.0822±0.0044	1.5653±0.5805	0.254±0.023		0.0296±0.0104
Male 2	V2	35	0.265±0.020	0.1733±0.0650	0.0837±0.0191	0.159±0.021		0.0634±0.0059
Male 3	V2	35	0.063±0.005	0.4886±0.0453	0.0113±0.0020	0.138±0.008	0.0000±0.0000	0.5175±0.1820
Female A	V4	35	0.748±0.040	0.0558±0.0033	0.0234±0.0121	0.162±0.015	0.0000±0.0000	0.2822±0.0447
Hybrid A1	V4	35	2.750±0.359	0.0185±0.0044	0.1956±0.0098	0.180±0.006		0.0155±0.0148
Hybrid A2	V4	35	1.060±0.174	0.0444±0.0110	0.0659±0.0110	0.171±0.003		0.0759±0.0354
Hybrid A3	V4	35	1.016±0.066	0.0085±0.0007	0.0051±0.0029	0.145±0.043	0.0000±0.0000	0.0600±0.0081
Male 1	V4	35	6.687±0.882	0.0050±0.0008	1.7546±0.2178	0.348±0.024		0.0615±0.0530
Male 2	V4	35	3.727±0.415	0.0010±0.0004	1.9667±0.0638	0.392±0.007		0.0029±0.0021
Male 3	V4	35	1.856±0.451	0.0207±0.0039	0.0567±0.0218	0.157±0.061	0.0000±0.0000	0.1054±0.0287
Female A	V8	35	4.354±0.221	0.0370±0.0120	0.4580±0.0059	0.134±0.042	0.0000±0.0000	0.0015±0.0015
Hybrid A1	V8	35	7.402±3.080	0.0062±0.0020	0.4535±0.0902	0.070±0.041		0.0020±0.0007
Hybrid A2	V8	35	5.282±0.426	0.0035±0.0015	1.0869±0.2596	0.062±0.002		0.0162±0.0124
Hybrid A3	V8	35	4.552±0.635	0.0030±0.0012	0.3687±0.0925	0.144±0.053	0.0000±0.0000	0.0029±0.0022
Male 1	V8	35	6.928±0.346	0.0008±0.0001	2.3866±0.2753	0.228±0.010		0.0010±0.0006
Male 2	V8	35	4.361±0.619	0.0000±0.0000	3.6885±0.2538	0.172±0.038		0.0000±0.0000
Male 3	V8	35	5.337±0.758	0.0026±0.0011	0.5876±0.3238	0.092±0.020	0.0000±0.0000	0.0000±0.0000

SE, standard error of the mean.

Supplementary Table 9. Means and standard error of relative gene expression for each genotype by treatment combination of Batch B.

Genotype	Treatment	Sampling Time	*VRN-H1*±SE	*VRN-H2*±SE	*VRN-H3*±SE	*PPD-H1*±SE	*PPD-H2*±SE	*HvODDSOC2*±SE
Female B	V2	17	0.003±0.001	0.1060±0.0063	0.0005±0.0003	0.084±0.001	0.0000±0.0000	0.0531±0.0136
Hybrid B1	V2	17	1.761±0.068	0.0807±0.0054	0.0050±0.0005	0.717±0.023		0.1247±0.0035
Hybrid B2	V2	17	0.005±0.002	0.1588±0.0086	0.0001±0.0000	0.130±0.001		0.1445±0.0263
Hybrid B3	V2	17	0.029±0.011	0.1448±0.0012	0.0005±0.0001	0.179±0.029	0.0000±0.0000	0.1111±0.0370
Male 1	V2	17	3.621±0.564	0.0340±0.0117	0.2950±0.0421	0.656±0.161		0.0051±0.0004
Male 2	V2	17	0.006±0.001	0.2690±0.0369	0.0043±0.0030	0.247±0.004		0.0102±0.0019
Male 3	V2	17	0.030±0.004	0.2114±0.0044	0.0002±0.0001	0.175±0.007	0.0000±0.0000	0.0796±0.0173
Female B	V4	17	0.267±0.061	0.0851±0.0061	0.0013±0.0006	0.156±0.018	0.0000±0.0000	0.1662±0.0339
Hybrid B1	V4	17	1.212±0.084	0.0235±0.0057	0.0077±0.0028	0.271±0.013		0.0386±0.0125
Hybrid B2	V4	17	0.640±0.018	0.1168±0.0068	0.0179±0.0018	0.294±0.009		0.3471±0.0094
Hybrid B3	V4	17	1.630±0.387	0.0769±0.0075	0.0240±0.0141	0.508±0.087	0.0014±0.0007	0.0222±0.0081
Male 1	V4	17	5.901±0.251	0.0172±0.0027	0.6981±0.0068	0.517±0.103		0.0008±0.0001
Male 2	V4	17	1.945±0.101	0.0208±0.0049	0.2501±0.0264	0.188±0.019		0.0037±0.0010
Male 3	V4	17	1.169±0.073	0.1108±0.0026	0.0218±0.0020	0.320±0.011	0.0218±0.0217	0.0927±0.0098
Female B	V8	17	3.376±0.795	0.0028±0.0015	0.2173±0.0820	0.159±0.055	0.0000±0.0000	0.0053±0.0032
Hybrid B1	V8	17	10.682±1.064	0.0156±0.0011	0.4715±0.0034	0.235±0.020		0.0074±0.0027
Hybrid B2	V8	17	7.879±1.405	0.0043±0.0012	0.5999±0.1565	0.239±0.020		0.0114±0.0012
Hybrid B3	V8	17	4.025±0.637	0.0053±0.0014	0.3453±0.1011	0.169±0.046	0.0040±0.0020	0.0007±0.0004
Male 1	V8	17	7.228±1.199	0.0129±0.0007	0.4734±0.0777	0.407±0.122		0.0010±0.0002
Male 2	V8	17	11.234±0.087	0.0005±0.0000	0.9459±0.0553	0.572±0.108		0.0013±0.0008
Male 3	V8	17	4.173±0.512	0.0139±0.0008	0.2158±0.0286	0.144±0.013	0.0088±0.0036	0.0010±0.0005
Female B	V2	35	0.068±0.022	0.2448±0.0354	0.0023±0.0009	0.213±0.044	0.0000±0.0000	0.0338±0.0050
Hybrid B1	V2	35	1.816±0.040	0.3362±0.0255	0.0313±0.0087	0.697±0.105		0.0579±0.0097
Hybrid B2	V2	35	0.043±0.013	0.3849±0.1273	0.0010±0.0003	0.146±0.030		0.0827±0.0475
Hybrid B3	V2	35	0.122±0.011	0.0894±0.0082	0.0023±0.0008	0.068±0.006	0.0000±0.0000	0.0800±0.0167
Male 1	V2	35	4.736±0.996	0.0278±0.0035	1.6047±0.1054	0.343±0.042		0.0031±0.0004
Male 2	V2	35	0.231±0.097	0.2535±0.0116	0.0851±0.0462	0.159±0.026		0.0027±0.0006
Male 3	V2	35	0.065±0.010	0.1498±0.0125	0.0018±0.0012	0.057±0.008	0.0000±0.0000	0.0242±0.0036
Female B	V4	35	1.135±0.026	0.0499±0.0055	0.1120±0.0017	0.284±0.007	0.0000±0.0000	0.0200±0.0016
Hybrid B1	V4	35	3.339±0.324	0.0101±0.0011	1.0726±0.2006	0.260±0.047		0.0010±0.0005
Hybrid B2	V4	35	4.001±0.161	0.0143±0.0029	0.4654±0.0687	0.292±0.015		0.1028±0.0150
Hybrid B3	V4	35	1.317±0.370	0.0336±0.0008	0.1076±0.0465	0.127±0.010	0.0000±0.0000	0.0188±0.0042
Male 1	V4	35	6.800±0.786	0.0346±0.0020	3.9462±0.5083	0.209±0.017		0.0001±0.0001
Male 2	V4	35	3.766±0.261	0.0006±0.0001	1.9697±0.0824	0.402±0.027		0.0008±0.0001
Male 3	V4	35	1.859±0.079	0.0193±0.0044	0.0801±0.0035	0.106±0.008	0.0034±0.0017	0.0369±0.0006
Female B	V8	35	6.196±1.271	0.0142±0.0027	3.5810±0.5596	0.187±0.043	0.0000±0.0000	0.0007±0.0007
Hybrid B1	V8	35	4.574±0.333	0.0157±0.0021	2.3064±0.5789	0.195±0.035		0.0012±0.0001
Hybrid B2	V8	35	5.705±0.602	0.0123±0.0030	3.4826±0.6685	0.270±0.015		0.0038±0.0006
Hybrid B3	V8	35	5.458±0.830	0.0109±0.0012	1.9484±0.4403	0.167±0.018	0.0761±0.0629	0.0138±0.0118
Male 1	V8	35	6.825±1.254	0.0139±0.0015	2.4662±0.3629	0.215±0.037		0.0001±0.0001
Male 2	V8	35	6.117±1.252	0.0014±0.0001	4.6541±1.3786	0.130±0.017		0.0004±0.0004
Male 3	V8	35	6.908±0.914	0.0165±0.0024	1.5454±0.3053	0.129±0.005	0.0008±0.0008	0.0045±0.0012

SE, standard error of the mean.
